# Structural and Biofunctional Insights into the Cyclo(Pro-Pro-Phe-Phe-) Scaffold from Experimental and In Silico Studies: Melanoma and Beyond †

**DOI:** 10.3390/ijms23137173

**Published:** 2022-06-28

**Authors:** Joanna Bojarska, Martin Breza, Milan Remko, Malgorzata Czyz, Anna Gajos-Michniewicz, Michał Zimecki, Krzysztof Kaczmarek, Izabela D. Madura, Jakub M. Wojciechowski, Wojciech M. Wolf

**Affiliations:** 1 Institute of General and Ecological Chemistry, Faculty of Chemistry, Lodz University of Technology, 116 Zeromskiego Street, 90-924 Lodz, Poland; wojciech.wolf@p.lodz.pl; 2 Department of Physical Chemistry, Slovak Technical University, Radlinskeho 9, SK-81237 Bratislava, Slovakia; martin.breza@stuba.sk; 3 Remedika, Luzna 9, SK-85104 Bratislava, Slovakia; milan.remko@gmail.com; 4 Department of Molecular Biology of Cancer, Medical University of Lodz, 6/8 Mazowiecka Street, 92-215 Lodz, Poland; malgorzata.czyz@umed.lodz.pl (M.C.); anna.gajos-michniewicz@umed.lodz.pl (A.G.-M.); 5 Institute of Immunology and Experimental Therapy, Polish Academy of Sciences, 12 Weigla Street, 53-112 Wroclaw, Poland; michal.zimecki@hirszfeld.pl; 6 Institute of Organic Chemistry, Faculty of Chemistry, Lodz University of Technology, 116 Żeromskiego Street, 90-924 Lodz, Poland; krzysztof.kaczmarek@p.lodz.pl; 7 Faculty of Chemistry, Warsaw University of Technology, 3 Noakowskiego Street, 00-664 Warsaw, Poland; izabela@ch.pw.edu.pl; 8 Rigaku Europe SE, Hugenottenallee 167, 63263 Neu-Isenburg, Germany; jakub.wojciechowski@rigaku.com

**Keywords:** short peptides, homo amino acids, melanoma, DFT, Hirshfeld surface, electrostatic potential, energy frameworks

## Abstract

Short peptides have great potential as safe and effective anticancer drug leads. Herein, the influence of short cyclic peptides containing the Pro-Pro-Phe-Phe sequence on patient-derived melanoma cells was investigated. Cyclic peptides such as cyclo(Leu-Ile-Ile-Leu-Val-Pro-Pro-Phe-Phe-), called CLA, and cyclo(Pro-homoPro-β^3^homoPhe-Phe-), called P11, exert the cytotoxic and the cytostatic effects in melanoma cells, respectively. CLA was the most active peptide as it reduced the viability of melanoma cells to 50% of control at about 10 µM, whereas P11 at about 40 µM after 48 h incubation. Interestingly, a linear derivative of P11 did not induce any effect in melanoma cells confirming previous studies showing that cyclic peptides exert better biological activity compared to their linear counterparts. According to in silico predictions, cyclic tetrapeptides show a better pharmacokinetic and toxic profile to humans than CLA. Notably, the spatial structure of those peptides containing synthetic amino acids has not been explored yet. In the Cambridge Structural Database, there is only one such cyclic tetrapeptide, cyclo((R)-β**^2^**homoPhe-D-Pro-Lys-Phe-), while in the Protein Data Bank—none. Therefore, we report the first crystal structure of cyclo(Pro-Pro-β^3^homoPhe-Phe-), denoted as 4B8M, a close analog of P11, which is crucial for drug discovery. Comparative molecular and supramolecular analysis of both structures was performed. The DFT findings revealed that 4B8M is well interpreted in the water solution. The results of complex Hirshfeld surface investigations on the cooperativity of interatomic contacts in terms of electrostatic and energetic features are provided. In short, the enrichment ratio revealed O^…^H/H^…^O and C^…^H/H^…^C as privileged intercontacts in the crystals in relation to basic and large supramolecular H-bonding synthon patterns. Furthermore, the ability of self-assemble 4B8M leading to a nanotubular structure is also discussed.

## 1. Introduction

According to the World Health Organization, cancer is still the leading cause of human deaths worldwide, either in high-, middle-, or low-income countries [1]. Melanoma, the most aggressive type of skin cancer, is responsible for ~80% of deaths in this class of malignancy, especially in young people under the age of 30 years [2,3]. Unfortunately, this incidence has risen globally, despite the recent progress in treatment [4,5,6,7]. One of the reasons is too late diagnosis. However, more importantly, tumor heterogeneity and innate and acquired resistance to drugs are key problems [5,8]. Therefore, there is still an urgent need to develop efficient therapeutic strategies, better delivery, and diagnostic systems. 

Short peptides, combining advantages of biologics and small chemical molecules, are promising effective and safe theranostics. Notably, they do not induce undesired autoimmune responses [9]. Ultra-short peptides have a better solid tumor penetration property and the ability to cross epithelial tissues [10,11,12]. Recent scientific and biotechnological progress as well as more advanced synthesis methods enabling sophisticated structural modifications help to overcome peptide shortcomings [12]. Generally, cyclic peptides have greater rigidity and consequently, stability towards exopeptidase hydrolysis due to the lack of carboxy and amino termini [13,14]. They have a large surface region providing high affinity and selectivity for protein targets. It is noteworthy that cyclic peptides can spontaneously form simple nanotubular assemblies through the intermolecular hydrogen-bond network. They have great potential for a wide range of bio-applications, including effective strategies for treatment in oncology and drug delivery to targeted tissues with low toxicity as well as early and more accurate diagnosis [15,16,17]. Short cyclic nanotubes are intriguing nanostructures due to their appealing features, such as simplicity, biocompatibility, high surface area, increased drug loading, environmental stability, enhanced permeation, and modifiable drug release [18,19,20,21]. 

It has been demonstrated that for some large peptides and related compounds even if they exert anticancer activity in preclinical studies, their translational value is low. Namely, kahalalide F, cyclic lariat tridecapeptide, or depsipeptides, containing the cyclic amino acid residues, such as proline (Pro) and phenylalanine (Phe), showed potent cytotoxic activity against solid tumors, including melanoma, and were enrolled in clinical trials, but the clinical studies were not successful due to inter alia toxicity and poor targeting [22,23]. However, cyclic oligopeptides are promising, and cytotoxicity of flaxseed linoorbitides with Pro and Phe in the amino acid sequence, against melanoma A375 cell lines, was observed [24]. On the other hand, these compounds can be toxic to humans, especially as intravenous infusion [24], but other formulations may be considered in the treatment of melanoma as well as other types of skin cancers [24]. Thus, further studies on synthetic derivatives of cyclic oligopeptides are required. 

According to our previous investigations, the Pro-Pro-Phe-Phe sequence in parent nonadeca cyclolinopeptide A (CLA) and its analogs play a pivotal role in bioactivity [25]. Moreover, proline has a significant impact on anticancer activity, and cyclic peptides containing aromatic amino acid residues possess extra rigidification of the backbone leading to increasing affinity to diverse targets [26,27]. Furthermore, synthetic amino acids such as β-homo amino acid residues can improve pharmacological properties, mainly bioactivity and stability, in vivo half-life, lipophilicity, potency, selectivity, and reduced toxicity [28].

Hence, in this study, the utility of short peptides based on the Pro-Pro-Phe-Phe scaffold, and their analogs with synthetic amino acids, was tested against melanoma. More specifically, cyclo(Leu-Ile-Ile-Leu-Val-Pro-Pro-Phe-Phe-), denoted as CLA; cyclo(Pro-Pro-β^3^homoPhe-Phe-), as 4B8M; cyclo(Pro-Pro-β^3^homoPhe-Phe-Pro-Pro-β^3^homoPhe-Phe-), 4B8D; cyclo(Pro-homoPro-β^3^homoPhe-Phe-), as P11; H-Pro-homoPro-β^3^homoPhe-Phe-OH, P11L; H-D-Pro-Pro-β^3^homoPhe-Phe-OH, as P03L, H-Pro-Pro-D-β^3^homoPhe-Phe-OH, as P04L were investigated. In addition, cyclo(Phe-Phe-), denoted as DKP1, was included in the analysis because of the proven anticancer activity of cyclic dipeptides, including melanoma [29,30]. 

It can be mentioned that among the studied peptides, 4B8M has been best characterized by its in vitro and in vivo activities, as well as the mechanism of action, predominantly in the mouse model [31]. Apart from its wide spectrum of anti-inflammatory properties [31] the peptide was recently shown to exhibit antiviral activities [32]. It inhibits replication of human adenovirus C serotype 5 and herpes simplex virus type 1 [32]. 

Moreover, our findings revealed that 4B8M peptide inhibits mitogen-stimulated proliferation of mouse splenocytes. Discussion of results, statistics and figures are presented elsewhere [31].

The mechanism of action of 4B8M has been associated with the regulation of prostanoid metabolism [31] and the most important finding was, relevant to this study, that the peptide selectively inhibited lipopolysaccharide-induced expression of prostaglandin E2 (PGE2) receptors, such as EP1 and EP3. The role of EP1/EP3 receptors in PGE2-mediated tumor differentiation and progression [33,34], as well as tumor lymphangiogenesis [35] is known. It also appears that inhibition of topical tumor growth by the antitumor drug apigenin was also associated with a decrease in EP1 receptors [36]. A property of 4B8M to inhibit the expression of an adhesion molecule ICAM-1 [31] may also negatively affect the progression of tumors. 

It should be also mentioned that hydrophobic compounds have a high potential to cross the cellular membrane. Cyclo-peptides exert special therapeutic capacity not only due to resistance to proteolysis but also due to increased cell membrane permeability. 4B8M was proven to be very effective when applied in an ointment for the amelioration of inflammatory skin changes [31] demonstrating its ability to penetrate the corneal (epithelial) skin layer. This peptide has a low molecular weight and as a potential drug has no obstacle to enter cells. In our previous studies, we observed that 4B8M was of low toxicity towards resident mouse and human cells, and reference cell lines [31,32]. Specifically, at 50 µg/mL, the cytotoxicity for mouse splenocytes of CLA and 4B8M was 50% and 15%, respectively [31]. Lack of toxicity against the epithelial lung cell line A-549 and towards proliferating splenocytes was also observed [32].

Moreover, to complement the analysis, we checked the influence of β-homo-Pro/Phe on the bioactive potential of synthesized short cyclo-peptides. In this context, we tested compounds using diverse web-based in silico tools to give a preliminary insight into pharmacological, toxic, drug-likeness, and target metrics. 

Another interesting issue seemed to be the fact that the spatial structure of cyclo-tetrapeptides containing homo/β amino acids is unexplored yet. The only such structure deposited in the Cambridge Structural Database (CSD, Version 5.43, last updated November 2021) [37,38] is the X-ray crystal structure of a (13-membered) cyclo-tetrapeptide with unnatural amino acids cyclo((R)-β-phenylalanyl-D-prolyl-(N-t-methoxycarbonyl)lysyl-phenylalanyl; cyclo(-(R)-β**^2^**homoPhe-D-Pro-Lys-Phe), CSD reference code YEPVIZ [39]. It can be mentioned that in the CSD we found derivatives of the decapeptide antamanide [40,41,42] as the only peptide structures containing the Pro-Pro-Phe-Phe motif, while in the Research Collaboratory for Structural Bioinformatics Protein Data Bank (RCSB PDB) [43]—none, despite the increasing number of crystal structures of short cyclo-peptides deposited in both structure databases recently. Notably, the determination of the three-dimensional (3D) structure is essential either for rational drug design and development or for the identification of new targets. Therefore, here we present the first model crystal structure of tetrapeptide cyclo(Pro-Pro-β^3^homoPhe-Phe-), called 4B8M, [IUPAC name: (3S,9S,13S,16S)-9,13-dibenzyl-1,7,10,14-tetraazatricyclo[14.3.0.03,7]nonadecane-2,8,11,15-tetrone], together with comparative ancillary analysis towards YEPVIZ [39], in line with our interest on (supra)molecular exploration of peptide-based molecules [11,12,25,29,44,45,46,47,48,49,50,51,52,53,54,55,56,57,58,59,60,61,62,63,64,65,66,67,68,69,70,71,72,73,74,75,76]. In this context, the results of complex Hirshfeld surface investigations on interatomic contacts, electrostatic and energetic features, as well as density functional theory (DFT) are discussed. Those in silico structural studies could be crucial in the drug discovery process. More specifically, Hirshfeld surface maps and the molecular electrostatic potentials characterize and visualize molecular shapes complementarity of potential ligands and their intercontact surfaces related to neighboring molecules providing valuable information on the molecular environment, the topology of interactions, and binding affinity. The electrostatic potential mapped on the Hirshfeld surfaces can be helpful in the identification of a 3D ‘pharmacophore’ model surface showing hydrogen-bond acceptors and donors [77] as well as key hydrogen-bonding patterns, the so-called synthons, that are a blueprint of rational design and development of more effective drugs [78,79,80].

Furthermore, the increased rigidity of those cyclopeptide backbones that ensure the formation of nanotubes via the supramolecular interactions is also worthy of note [81]. Thus, the ability of self-organization/self-assembling of this cyclotetrapeptide, leading to the nanotubular structure, was investigated too. 

## 2. Results and Discussion

### 2.1. Biological Studies

In this study, melanoma cell lines derived from surgical specimens were used. Cell lines were named DMBC28 and DMBC29 after the Department of Molecular Biology of Cancer. They harbor a heterozygous mutation leading to BRAF^V600E^, and the NF1^R135W^ variant has been found in DMBC28 cells [82]. Extensive analysis of whole-exome sequencing data for DMBC28 and DMBC29 cells revealed several mutations in addition to above mentioned [82], as shown in the Electronic Supporting Information (Tables S1 and S2). At the phenotype level, both cell lines exert high expression of MITF-M, a lineage-specific transcription factor, and low activity of ERK1/2, an effector kinase of the MAPK pathway [83,84]. Transcript levels of SOX2 and AXL are below the median value calculated for other melanoma cell lines, and the percentages of cells expressing the nerve growth factor receptor (NGFR), a marker of a more primitive phenotype, are high in DMBC28 cells and low in DMBC29 cells in comparison to other melanoma cell lines [85]. Transcript levels of MLANA, PMEL, TYR, and DCT are markedly higher in DMBC29 and DMBC28 cells, in comparison to the median values calculated for other melanoma cell lines [86]. These two cell lines have been recently used to investigate the mechanism of melanoma cell death in response to encorafenib, a recently FDA-approved inhibitor of melanoma with BRAF^V600^ [86].

In the current study, we focused on the influence of short peptides, cyclic and their linear analogs, on the viability of DMBC29 and DMBC28 melanoma cells. For that, three different methods were applied assessing either only viable cells after treatment with short peptides or both the percentages of viable and dead cells were evaluated. In addition, peptide-induced changes in cell morphology were analyzed.

Descriptions of all analyzed compounds are summarized in Table S3.

#### 2.1.1. P11 and CLA Decreases Cell Confluence in Concentration and Time Dependent Manner

**Figure 1:**
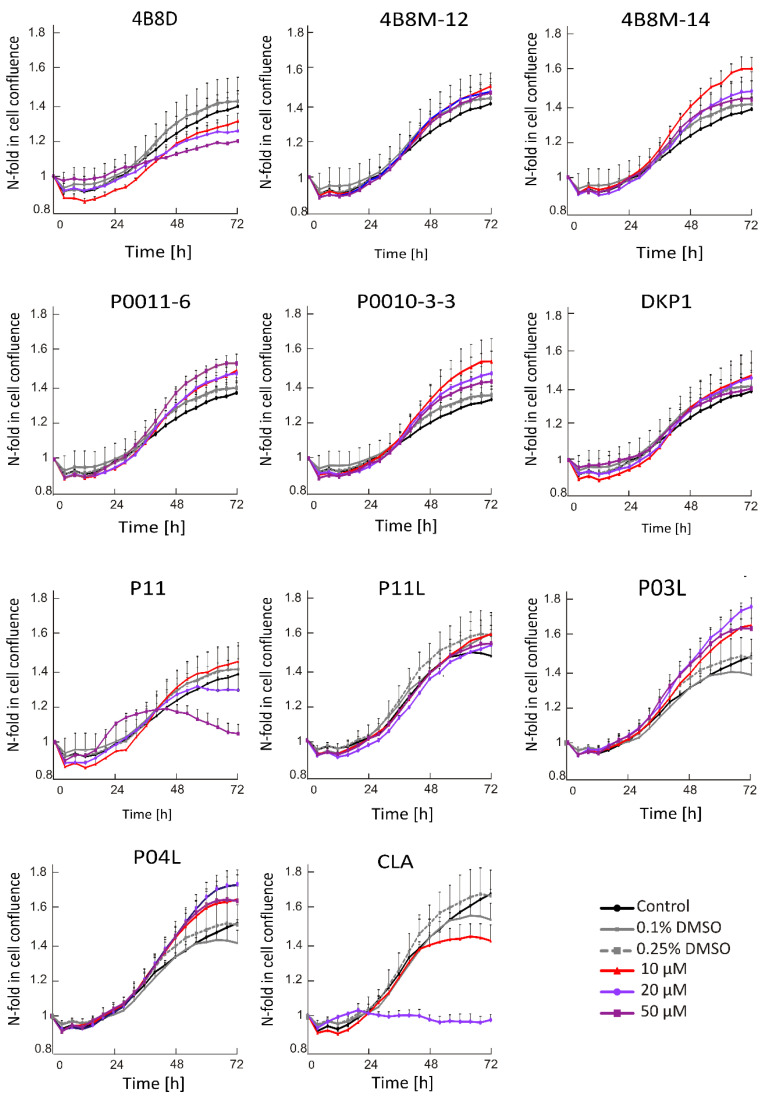
Time–course curves for short peptide-treated or vehicle-treated DMBC29 cells (0.1%; 0.25% dimethyl sulfoxide, DMSO). Control stands for melanoma cells cultured without short peptides or vehicle. Changes in the occupied area (% confluence) over time are shown as fold-change relative to the confluence at time T_0_. Plots represent mean values of representative experiment performed in triplicates ± SD.

Time-lapse microscopy (IncuCyte ZOOM) was used to measure real-time changes in cell confluence during treatment. Time–course curves obtained for cells exposed either to a vehicle or to short peptides at indicated concentrations are shown in Figure 1. There were no inhibitory effects on cell confluence after the treatment of DMBC29 cells with short peptides, except for P11 and CLA. The strongest effect was observed for CLA since this peptide already at 20 μM completely restrained an increase in cell confluence. At this concentration, the other peptide only slightly reduced an increase in cell confluency over time; however, when P11 was used at 50 μM, a substantial but delayed effect was detected.

#### 2.1.2. P11 and CLA Efficiently Decrease Number of Viable Cells in APA Assay

**Figure 2:**
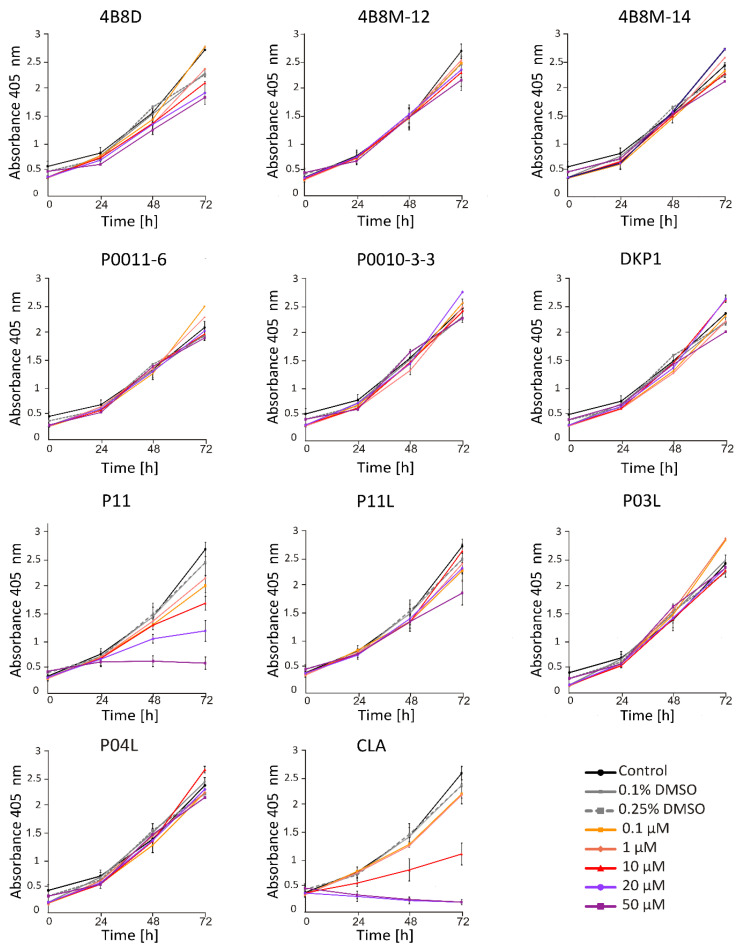
The influence of short peptides or a vehicle (0.1%; 0.25% DMSO) on viability of DMBC29 cells based on changes in their metabolic activity (APA assay). Control stands for melanoma cells cultured without short peptides or vehicle. Data presented are mean ± SD; n = 3 for 4B8M-12, P11, P11L and CLA and as mean ± SD; n = 2 for the rest of tested compounds. Statistically significant differences are shown in the Electronic Supporting Information (Figure S1A).

**Figure 3:**
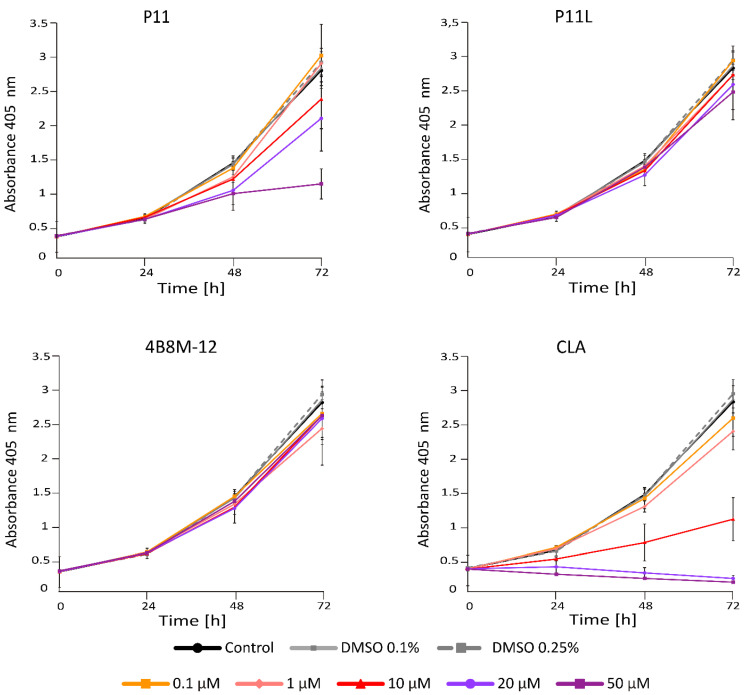
The influence of selected short peptides or a vehicle (0.1%; 0.25% DMSO) on viability of DMBC28 cells based on changes in their metabolic activity (APA assay). Control stands for melanoma cells cultured without short peptides or vehicle. Data presented are mean ± SD; n = 3. Statistically significant differences are shown in Figure S1B.

Acid Phosphatase Activity (APA) assay provides a readout of cell status through measurement of metabolic activity of cells. This method enabled the assessment of the viable melanoma cell number at indicated time points following incubation with short peptides. To investigate the activity of short peptides, we first determined their activity at different concentrations. It has been shown that at 20 μM, P11 and CLA substantially reduced the number of viable DMBC29 cells after 48 h and 24 h of treatment, respectively, whereas other peptides, including P11L (a linear derivative of P11), did not influence the melanoma cell viability (Figure 2). Of note, CLA reduced the number of viable cells already at 10 μM (Figure 2). Similar results were obtained when the influence of selected peptides was assessed in DMBC28 cells (Figure 3). 

**Figure 4:**
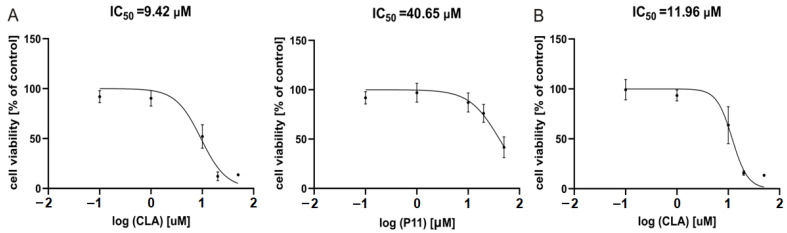
Dose response curves for melanoma cells exposed to CLA and P11 for 48 h. (Panel **A**) shows response curves for DMBC29 cells, (panel **B**) for DMBC28 cells. IC_50_ values are indicated. n = 3.

Based on results obtained after 48 h of incubation with peptides, IC_50_ values for the most active peptides, P11 and CLA, were calculated with the function ‘log [inhibitor] vs. normalized response’ (Graph Pad Prism9). As shown in Figure 4A, IC_50_ values displaying effects in DMBC29 cells were 9.42 μM and 40.65 μM for CLA and P11, respectively. For DMBC28 cells, the IC_50_ value calculated for CLA-treated cells was 11.96 μM (Figure 4B), whereas calculation of the IC_50_ value for P11 was possible only after 72 h incubation for the range of concentrations used in this study, and was 44.9 μM (not shown). 

#### 2.1.3. CLA Effectively Induces Cell Death in Concentration Dependent Manner

**Figure 5:**
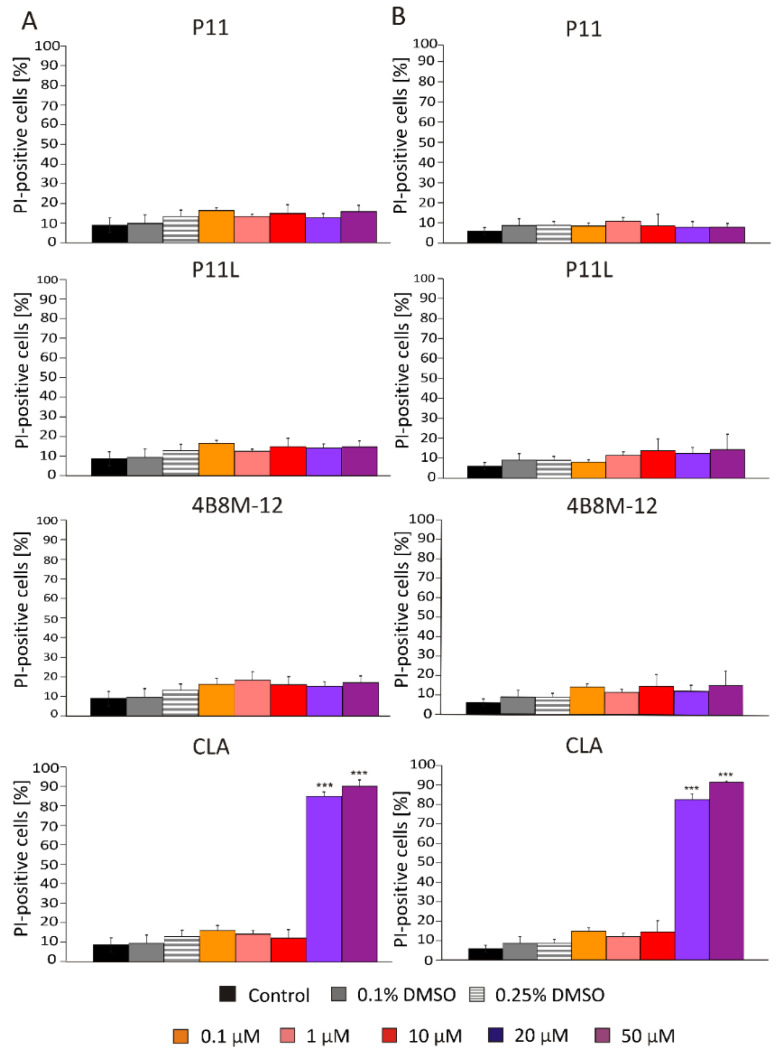
Percentages of dead melanoma cells (PI-positive) in DMBC29 cells (panel **A**) and DMBC28 cells (panel **B**) after 48 h of treatment with selected short peptides. Bars represent mean values of three biological replicates ± SD. Statistically significant differences are indicated as *** *p* < 0.001.

To provide a direct identification of viable and dead cells in a cell population after treatment with short peptides, flow cytometry was applied. Propidium iodide (PI) was used to identify dead cells (PI-positive) with damaged membranes. Obtained results show that only CLA induced cell death, already at 20 μM in DMBC29 cells (Figure 5, panel A) and DMBC28 cells (Figure 5, panel B). P11, P11L, and 4B8M-12 did not increase the percentages of PI-positive cells. 

#### 2.1.4. Assessment of Changes in Cell Morphology after Exposure to Short Peptides

**Figure 6:**
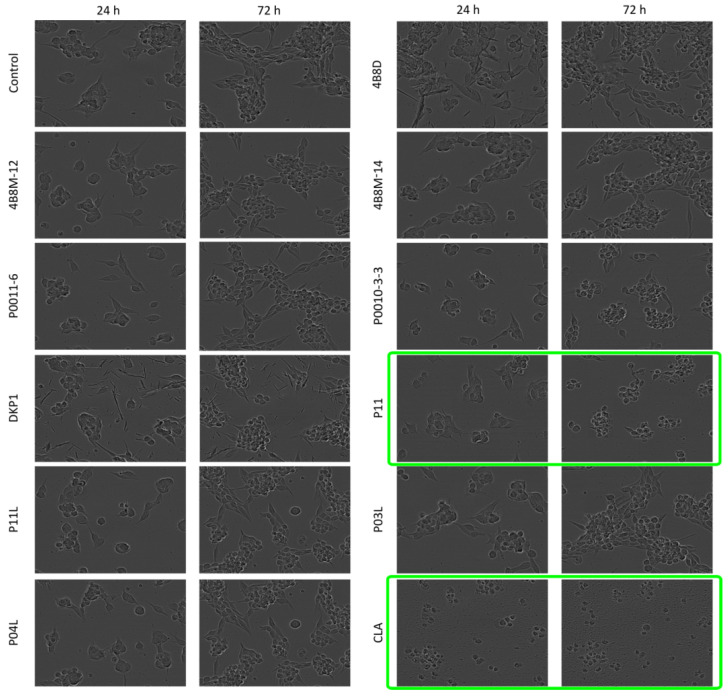
Microphotographs of DMBC29 cells exposed to short peptides at 50 μM for 24 h and 72 h. Microphotographs showing cell death are green-framed.

Morphological changes such as cell rounding were observed in P11- and CLA-treated DMBC29 cells. Cells lost their adherent phenotype when they were exposed to 50 μM P11 for 72 h or to 20 μM CLA already after 24 h of exposure. Cell death was detected in the culture of DMBC29 cells after 48 h exposure to 20 μM CLA and already after 24 h to 50 μM CLA (Figure 6 and Figure S2). Precipitation of 4B8D and DKP1 in the culture medium occurred and is visible in the microphotographs. No substantial changes in cell morphology were induced by other short peptides (Figure S2).

To conclude, the study was performed on patient-derived melanoma cells that were cultured in stem cell medium. It has been found that P11 exerted a cytostatic effect inhibiting cell proliferation without inducing cell death, whereas CLA exerted a cytotoxic effect on DMBC29 cells. Similar effects were observed in DMBC28 cells treated with P11 and CLA; however, the cytostatic effect of P11 was observed after 48 h at a higher concentration (50 μM). Analysis of cell confluence and morphological changes in DMBC29 cells treated with short peptides confirmed the activity of P11 (loss of adherent cells at 50 μM) and CLA (shrinkage and disassembly of cells at 20 μM). P11L, the linear variant of P11, as well as 4B8M-12, did not exert any substantial influence on melanoma cell lines. 

### 2.2. Molecular and Crystal Structure 

#### 2.2.1. Geometry Optimization

X-ray crystal structures are determined in the solid state where molecular objects are restrained by numerous interactions. On the hand, chemical reactions are usually realized in solution or solution-like environments where flexibility and dynamics of reacting species can be fully demonstrated. In those circumstances, the results of X-ray studies should be used with care. 

On the other hand, spectroscopic methods offer only partial information on the structures of molecules/ions in solutions. In some cases, significantly different properties of the same compound in crystals and solutions are observed. Fortunately, recent quantum-chemical methods are capable of fully describing the studied structures in solutions with sufficient accuracy via their geometry optimization. Moreover, such treatment provides information on the electronic structure (such as atomic charges and molecular orbitals) and related properties of the systems under study. 

**Figure 7:**
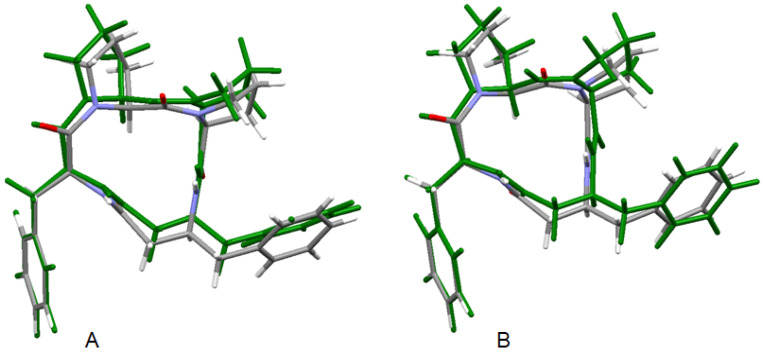
Superposition of the X-ray structure of 4B8M and the B3LYP optimized 4B8M in gas phase (**A**) and B3LYP optimized solvated 4B8M (**B**). The optimized structures are in green.

Geometry optimization calculations were performed using the hybrid B3LYP functional, which describes the molecular structure of organic compounds with high precision [87]. Harmonic vibrational frequencies calculations of the optimized conformers computed in both environments confirmed that all of them correspond to minima of potential energy surfaces (no imaginary vibrations). The starting structures for use in DFT geometry optimizations were obtained from X-ray crystallography data [39]. The optimized structures in the gas phase and in solution together with the solid-state X-ray structure 4B8M are shown in Figure 7 and their relevant structure data in Table 1. The large differences in the geometry of the central cyclic tetrapeptide moiety between the gas-phase and solid-state structures of these molecules can be explained by an environmental effect of crystal-packing forces. The solid-state 3D structure of the molecule is governed by a system of intermolecular hydrogen bonds of neighboring molecules, which is manifested especially at different torsion angles of the cyclic moiety. Structurally rigid phenyl rings are positioned almost equally in both environments. As regards the 4B8M molecule, its crystal structure is well interpreted in the water solution and its structural shapes in both environments are close to each other. 

The molecular superposition of the X-ray structure of YEPVIZ [39] and the corresponding gas phase and condensed phase (water) structures are shown in Figure S3 and their relevant structure data in Table S4. In the presence of intramolecular hydrogen bonds between the structural units, the equilibrium gas phase geometry of YEPVIZ closely mimics the experimental X-ray structure of this species in the solid state [39]. However, extensive intermolecular hydrogen bonds and crystal-packing forces govern the geometry in the solid state and result in different geometry parameters of its long aliphatic side chain containing polar groups. The effect of the environment (water) was accessed using the CPCM method. Water has a slight effect on the geometry of the cyclic tetrapeptide moiety, and this is close to that determined for the solid state. Larger differences in the structural arrangement were observed for the flexible aliphatic substituent only.

The solvent effect on both compounds under study is illustrated by their atomic charges (Table 2 and Table S5). As indicated by the differences between the vacuum and aqueous solution data, the maximal changes can be observed at oxygen and nitrogen atoms. Negative oxygen charges increase in the polar solvent, unlike nitrogen atoms, which become less negative. Only carbon atoms bonded to oxygen atoms have positive charges.

**Figure 8:**
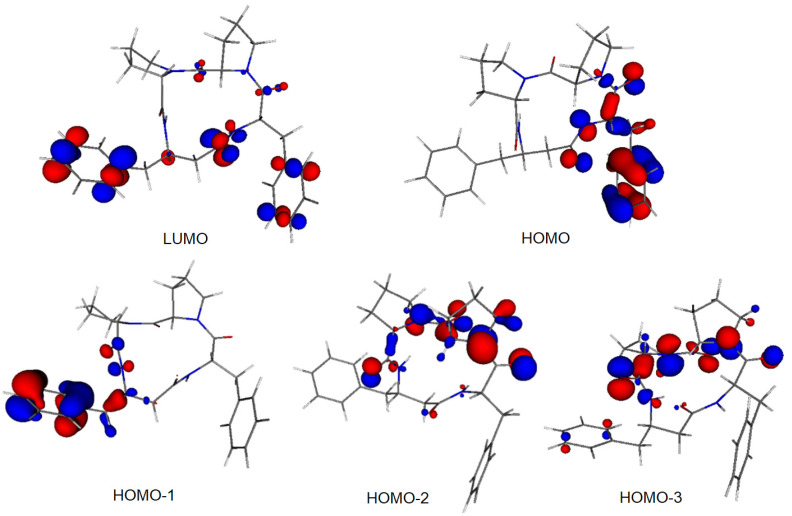
Frontier MOs of 4B8M (0.05 a.u. isosurface).

Molecular orbitals (MOs), especially the highest occupied molecular orbital (HOMO) and the lowest unoccupied molecular orbital (LUMO), are of crucial importance for molecular reactivity. The two highest occupied MOs, i.e., HOMO and HOMO-1, of both compounds are located prevailingly at one of the phenyl rings and at neighboring heteroatoms (Figure 8 and Figure S4). The LUMO of 4B8M is located in both phenyl rings and at its tetrapeptide ring whereas the LUMO of YEPVIZ is located at its tetrapeptide ring. The LUMO energies of both compounds are negative (Table S6) which indicates their high electron-acceptor ability. Solvent effects shift the LUMO energies to less negative values. On the other hand, the energies of occupied MOs are more negative in polar solvents. Nevertheless, the HOMO-LUMO energy gaps increase in the solvent, which indicates their higher stability in aqueous solutions.

**Table 1:** Relevant structure data of the tetrapeptide ring in 4B8M (see Figure 9 for atom notation).

Bond Length [Å]	X-ray	B3LYP	
		vacuum	solution
N1^—^C4	1.471 (3)	1.461	1.465
C4^—^C5	1.548 (3)	1.557	1.556
C5^—^N2	1.358 (3)	1.373	1.365
N2^—^C9	1.472 (3)	1.474	1.475
C9^—^C10	1.511 (4)	1.539	1.537
C10^—^N3	1.335 (3)	1.362	1.350
N3^—^C11	1.449 (3)	1.461	1.463
C11^—^C19	1.539 (3)	1.539	1.540
C19^—^C20	1.515 (3)	1.523	1.522
C20^—^N4	1.361 (4)	1.372	1.363
N4^—^C29	1.455 (3)	1.460	1.459
C29^—^C30	1.533 (3)	1.548	1.551
C30^—^N1	1.345 (2)	1.363	1.355
N1^—^C1	1.476 (3)	1.478	1.481
C1^—^C2	1.532 (3)	1.540	1.539
C2^—^C3	1.533 (3)	1.539	1.538
C3^—^C4	1.534 (3)	1.550	1.549
N2^—^C6	1.474 (3)	1.475	1.480
C6^—^C7	1.512 (4)	1.530	1.528
C7^—^C8	1.515 (5)	1.537	1.536
C8^—^C9	1.545 (3)	1.559	1.559
C5^—^O1	1.221 (3)	1.223	1.229
C10^—^O2	1.230 (4)	1.218	1.230
C20^—^O3	1.227 (4)	1.220	1.229
C30^—^O4	1.231 (3)	1.226	1.234
**Bond angle [°]**			
C30^—^N1^—^C4	130.2 (2)	131.3	130.8
N1^—^C4^—^C5	108.8 (2)	110.1	109.8
C4^—^C5^—^N2	117.8 (2)	118.3	118.5
C5^—^N2^—^C9	127.0 (2)	128.4	127.8
N2^—^C9^—^C10	115.7 (2)	115.0	114.9
C9^—^C10^—^N3	117.6 (2)	115.1	115.8
C10^—^N3^—^C11	122.5 (2)	123.7	125.1
N3^—^C11^—^C19	109.6 (2)	108.8	108.7
C11^—^C19^—^C20	109.9 (2)	110.8	110.9
C19^—^C20^—^N4	114.6 (2)	116.2	116.2
C20^—^N4^—^C29	119.9 (2)	120.9	122.0
N4^—^C29^—^C30	114.0 (2)	113.6	113.3
C29^—^C30^—^N1	120.1 (2)	120.1	120.3
C1^—^N1^—^C30	118.6 (2)	117.5	118.2
C4^—^N1^—^C1	109.8 (2)	111.2	110.9
N1^—^C1^—^C2	105.1 (2)	104.5	104.7
C1^—^C2^—^C3	104.3 (2)	104.6	104.7
C2^—^C3^—^C4	102.6 (2)	102.9	102.9
C3^—^C4^—^N1	101.3 (2)	101.1	101.3
C3^—^C4^—^C5	108.0 (2)	109.7	109.7
C5^—^N2^—^C6	120.4 (2)	120.0	120.2
C9^—^N2^—^C6	110.3 (2)	111.4	111.2
N2^—^C6^—^C7	102.7 (2)	102.5	102.5
C6^—^C7^—^C8	101.4 (2)	103.6	103.6
C7^—^C8^—^C9	104.7 (2)	105.0	104.8
C8^—^C9^—^N2	103.2 (2)	104.2	104.3
C8^—^C9^—^C10	108.9 (2)	109.8	110.0
C4^—^C5^—^O1	120.5 (2)	120.6	120.2
N2^—^C5^—^O1	121.5 (2)	120.9	121.2
C9^—^C10^—^O2	117.4 (3)	119.8	119.4
N3^—^C10^—^O2	124.9 (2)	125.0	124.8
C19^—^C20^—^O3	122.5 (2)	121.3	121.3
N4^—^C20^—^O3	122.8 (3)	122.5	122.5
C29^—^C30^—^O4	119.9 (2)	119.6	119.5
N1^—^C30^—^O4	119.8 (2)	120.1	120.0
**Torsion angle [°]**			
N1^—^C4^—^C5^—^N2	169.4 (2)	179.1	176.5
C4^—^C5^—^N2^—^C9	−1.9 (3)	−12.7	−9.6
C5^—^N2^—^C9^—^C10	−82.6 (3)	−75.8	−78.0
N2^—^C9^—^C10^—^N3	−10.8 (3)	−18.7	−17.8
C9^—^C10^—^N3^—^C11	177.3 (2)	170.9	172.5
C10^—^N3^—^C11^—^C19	−114.8 (2)	−118.7	−117.9
N3^—^C11^—^C19^—^C20	42.2 (3)	57.6	56.6
C11^—^C19^—^C20^—^N4	−120.3 (2)	−127.2	−127.2
C19^—^C20^—^N4^—^C29	−163.5 (2)	−167.6	−170.5
C20^—^N4^—^C29^—^C30	−136.6 (3)	−137.1	−138.5
N4^—^C29^—^C30^—^N1	59.8 (3)	59.2	62.5
C29^—^C30^—^N1^—^C4	−3.3 (3)	−9.6	−10.0
C30^—^N1^—^C4^—^C5	−113.6 (2)	−97.6	−100.6
C29^—^C30^—^N1^—^C1	161.4 (2)	169.3	165.1
C30^—^N1^—^C1^—^C2	−155.9 (2)	−166.7	−164.4
N1^—^C1^—^C2^—^C3	14.7 (2)	13.4	14.0
C1^—^C2^—^C3^—^C4	−34.4 (2)	−32.6	−32.8
C2^—^C3^—^C4^—^N1	40.8 (2)	39.1	39.0
C2^—^C3^—^C4^—^C5	−73.5 (2)	−77.1	−77.0
C4^—^C5^—^N2^—^C6	159.3 (2)	160.9	160.0
C5^—^N2^—^C6^—^C7	−135.5 (2)	−145.3	−142.5
N2^—^C6^—^C7^—^C8	−40.8 (3)	−36.3	−36.6
C6^—^C7^—^C8^—^C9	38.9 (3)	30.9	31.9
C7^—^C8^—^C9^—^N2	−21.8 (3)	−13.5	−14.7
C7^—^C8^—^C9^—^C10	−145.3 (2)	−137.1	−138.4
C8^—^C9^—^C10^—^N3	104.9 (3)	98.3	99.4
N1^—^C4^—^C5^—^O1	−16.2 (3)	−5.9	−8.4
C9^—^N2^—^C5^—^O1	−176.2 (2)	172.3	175.3
N2^—^C9^—^C10^—^O2	172.5 (2)	163.7	164.0
C11^—^N3^—^C10^—^O2	−6.3 (4)	−11.7	−9.5
C11^—^C19^—^C20^—^O3	57.0 (3)	50.8	51.1
C29^—^N4^—^C20^—^O3	19.2 (3)	14.4	11.2
N4^—^C29^—^C30^—^O4	−125.0 (2)	−126.2	−123.2
C4^—^N1^—^C30^—^O4	−178.5 (2)	175.8	175.7

**Table 2:** NBO atomic charges of 4B8M (see Figure 9 for atom notation).

Atom	Vacuum	Solution
O4	−0.632	−0.684
O1	−0.628	−0.670
N1	−0.497	−0.485
N2	−0.546	−0.531
O3	−0.630	−0.688
N4	−0.643	−0.630
O2	−0.617	−0.688
N3	−0.652	−0.635
C5	0.689	0.700
C1	−0.167	−0.165
C28	−0.207	−0.209
C3	−0.375	−0.376
C24	−0.185	−0.203
C4	−0.094	−0.091
C23	−0.033	−0.032
C22	−0.401	−0.401
C27	−0.203	−0.205
C29	−0.096	−0.091
C30	0.693	0.704
C14	−0.204	−0.205
C20	0.688	0.699
C9	−0.088	−0.089
C12	−0.392	−0.400
C26	−0.208	−0.218
C2	−0.399	−0.399
C8	0.682	0.691
C6	−0.165	−0.166
C13	−0.031	−0.032
C19	−0.458	−0.461
C25	−0.191	−0.206
C18	−0.188	−0.206
C16	−0.207	−0.217
C15	−0.201	−0.205
C11	−0.021	−0.011
C17	−0.189	−0.205
C7	−0.390	−0.390
C8	−0.376	−0.374

#### 2.2.2. X-ray Diffraction Studies

**Figure 9:**
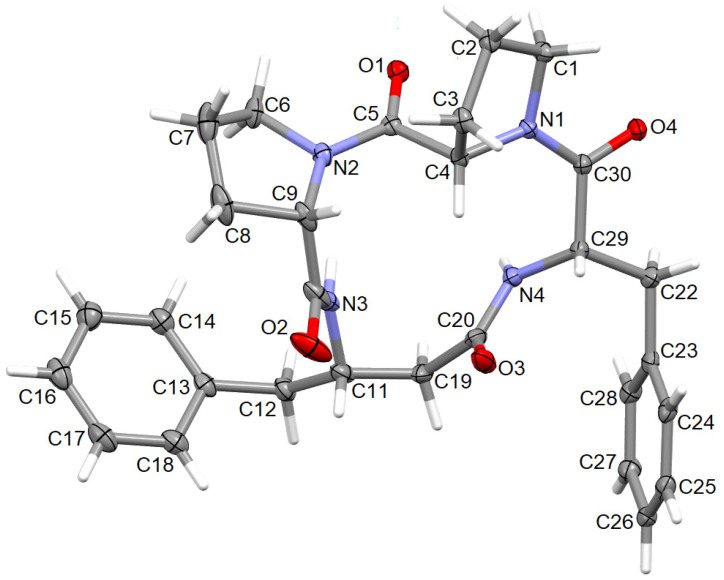
The molecular structure of 4B8M, showing the atom-labeling scheme. Displacement ellipsoids are drawn at the 30% probability level and H atoms are shown as small spheres of arbitrary radii.

**Figure 10:**
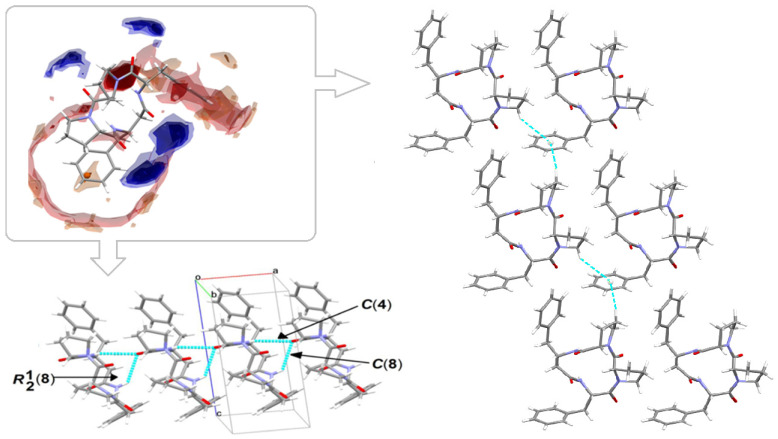
On the left in the frame: full interaction map of 4B8M showing the suitability of the interactions adopting the intensified red (H-bond acceptors) and blue regions (H-donors). On the left bottom and on the right: predicted interactions involved in the supramolecular synthons leading to 3D network.

The perspective view of the model molecular structure of 4B8M with the non-hydrogen atom labeling scheme is presented in Figure 9. The molecule has four chiral centers, namely *S* at C4, C9, C11, and C29. Benzene rings are planar. The Cremer–Pople parameters suggest half-chair conformation of the N1-C1-C2-C3-C4 proline ring [*φ* = 122.4 (3)^°^] and the envelope for N2-C6-C7-C8-C9 proline ring [292.1 (4) **^°^**] [88]. Peptide bonds have a planar trans conformation. X-ray crystallographic analysis revealed that the model compound crystallizes in the triclinic space group P1, a = 5.6034 (2), b = 9.9007 (3), c = 12.5815 (4) Å, α = 67.264 (1), ß = 87.284 (1), ɣ = 77.831 (2) **^°^** and *Z* = 1. The full crystallographic data and refinement parameters are presented in Table 3. The selected bond lengths and torsion angles are given in Table S7 and, in general, these parameters are similar to other cyclo-oligopeptide crystal structures [37,38]. Geometrical data of H-bonds are collected in Table 4. The architecture of the crystal network is characterized by either intra- or intermolecular H-bonds between amine and carbonyl groups, such as N-H^…^N and C-H^…^O, in the range of 2.78 to 3.47 Å. In addition, C-H^…^π and C-O^…^π intercontacts are observed (Table S8). The latter plays a stabilizing role in the crystal lattice. The 3D full interaction map [89] of 4B8M presents the preferences of the intermolecular interactions by highlighting areas (maps) around the molecule with certain functional groups with probable intercontacts involved in the building of the supramolecular architecture. The red regions represent H-bond acceptors, while the blue region illustrates donors. The orange and brown fragments signify the aromatic interactions and hydrophobic nature, respectively. It is helpful in understanding the supramolecular hydrogen-bonding synthons [78,79,80]. Etter’s H-bond rules, based on electrostatic potentials, are useful in determining donor-acceptor interactions that lead to synthon patterns [90]. In particular, at the first level of graph-set theory, the supramolecular chain C(4) is formed by _(__NH)_N3-H3N^…^O2_(C=O)_, while C(8) through _(NH)_N4-H4N^…^O2_(C=O)_. Both interactions participate in the generation of rings R^1^_2_(8) (Figure 10). At the second level, C-H^…^O interactions are engaged in the building of additional chains and rings leading to a 3D H-bonding network. In YEPVIZ, apart from chains and rings, intramolecular rings (S-motifs) are observed, formed by both N-H^…^O and C-H^…^O hydrogen bonds (Figure S5). All basic synthons are collected as a library in Table S9.

#### 2.2.3. Long-Range Synthon Aufbau Modules 

**Figure 11:**
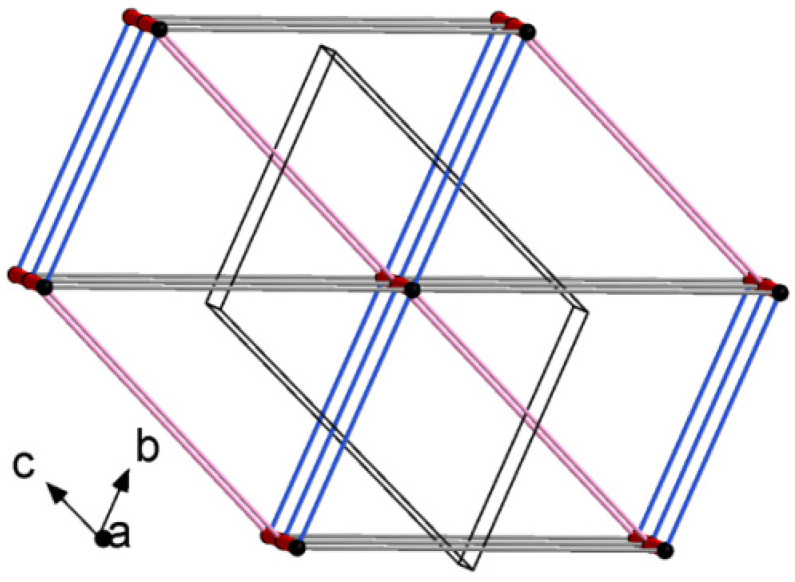
Large synthons in 4B8M: main C-H^...^O motifs in 4B8M; connectivity between molecules represented by their centers of gravity (black dots) via H-bonding.

**Figure 12:**
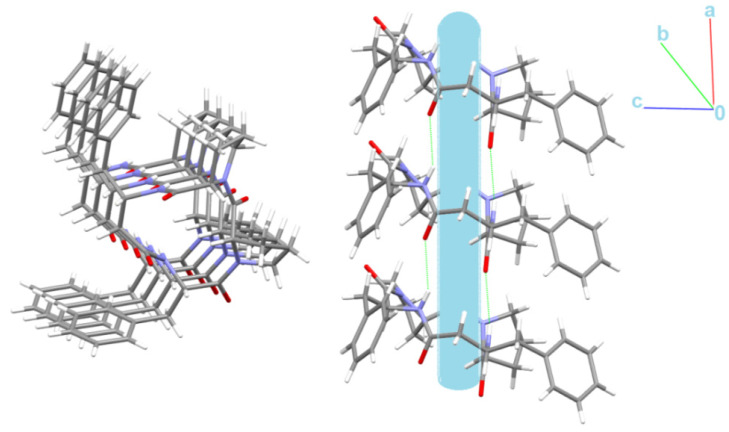
Nanotubular structure of 4B8M. Tube, with an internal diameter of ~2.5 Å, is presented in blue.

Schematic representations of long-range synthon Aufbau modules, so-called large synthons or LSAMs, making up parts of crystal structures and controlling the three-dimensionality of the structure [91], are shown in Figure 11 and Figure S6 for 4B8M and YEPVIZ, respectively. In 4B8M, the most important N-H^…^O bonds connect the molecules along the *[100]* direction into a 1D structure. The resulting tubes (Figure 12) exhibit the highest energy of intermolecular interactions and are packed almost hexagonally (Figure 11). There are directional C-H^...^O and C-H^...^π interactions between the tubes, but with the interaction energy three and five times lower than in the tube. Hence, in the 4B8M structure, the 1D motif should be treated as LSAM. Taking into account all directional interactions a uninodal 8-connected 3D network is formed, showing distorted bcu topology. Whereas, in the case of YEPVIZ, all the first-level motifs form a one-dimensional chain structure extending along the *[001]* direction (red tubes in the simplified packing diagram in Figure S6). In this 1D motif, the electrostatic energy component is dominant, hence it can be considered an LSAM. However, the chains do not pack hexagonally, which indicates the presence of other directional forces. There are weak C7-H10^…^O5 contacts between the chains, with the major share of dispersion energy, creating a double chain/ladder structure (pink lines in Figure S6). These ladders are almost hexagonally packed, and the distortion is mainly due to the presence of C23-H26^...^π interactions (thin gray lines in Figure S6). If we assume that the listed interactions are the most important in forming a crystal structure of YEPVIZ and the 1D chains are the LSAMs, the resulting 3D network might be considered a 5-connected unidonal one. However, if we consider the ladder as LSAMs the network simplifies to a 6-connected pcu (primitive cubic unit) topology (the vertex symbol 4^12^.6^3^), although with a mesh being a rhombus, not a square (Figure S6). 

Interestingly, the peptide C=O and NH groups are localized on the opposite sides of the backbone plane leading to columnar stacking of molecules linked by N-H^…^O intermolecular interactions, which try to form nanotubes with an internal diameter of ~2.5 Å (Figure 12). 

### 2.3. Hirshfeld Surface Analysis, and Enrichment Ratios

**Figure 13:**
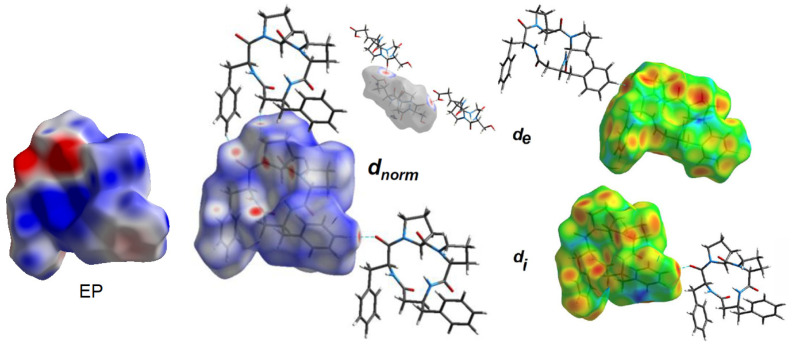
Electrostatic potential (EP) and Hirshfeld surface of 4B8M mapped with d_norm_, d_e_ and d_i_ properties.

**Figure 14:**
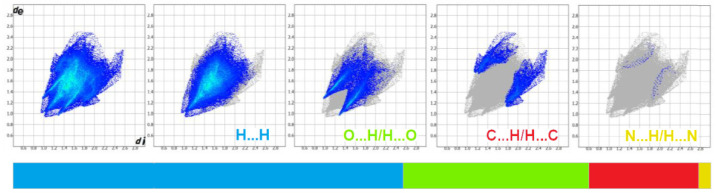
Fingerprint plots for 4B8M delineated into H^…^H, O^…^H/H^…^O, C^…^H/H^…^C, and N^…^H/H^…^N.

To obtain a deeper insight into the nature of interactions within the crystal of 4B8M and YEPVIZ, the topology of the molecular and interaction surfaces, electrostatic complementarity, using Hirshfeld surface analysis (HS) [92] were performed. The HS maps of 4B8M over d_norm_, d_e_, and d_i_ are visualized in Figure 13, while the HSs of YEPVIZ are presented in Figure S7. The dark-red areas on the d_norm_ surface demonstrate short intercontacts, while the light-red spots characterize the other interactions. The scheme of intercontacts is presented by the fingerprint plots (FP) (Figure 14 and Figure S8). The contribution of close contacts to the HS of both compounds is similar. At first glance, it is evident that H^…^H intercontacts are the main contributor. Most noticeably, O^…^H/H^…^O and C^…^H/H^…^C interactions contribute at the ~21% and 13% levels, respectively. The contribution of N^…^H/H^…^N interactions is mostly marginal (~0.5%). In YEPVIZ, O^…^H/H^…^O (15%) and C^…^H/H^…^C (12%) are crucial as well. What is more, the N^…^H/H^…^N contribution is still minimal (1.5%). 

The enrichment ratios (ER) of the intermolecular interactions in 4B8M and YEPVIZ based on the HS analysis are collected in Table S10. In this context, O^…^H/H^…^O and C^…^H/H^…^C are privileged intercontacts in the stability of both crystal structures. 

The molecular electrostatic potential (EP) [93] mapped over the HS of 4B8M signifies the electrostatic complementarity in the crystal packing of 4B8M about close intercontacts (Figure 13). In particular, the blue regions identify electropositive (donors), while the red areas signify electronegative (acceptors) parts. The charge parts correlate with the intercontacts visible on the d_norm_-, d_e_-, and d_i_-mapped HS. Detailed representations of EP over HS as well as all properties of HS of 4B8M and YEPVIZ are illustrated in Figure S7.

### 2.4. Energy Frameworks

**Figure 15:**
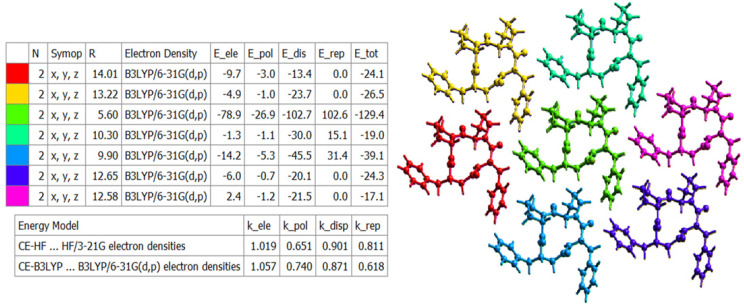
The color-coded mapping of interaction energies of the molecular pairs in terms of the energy frameworks of 4B8M (scale factors for benchmarked energy models).

**Figure 16:**
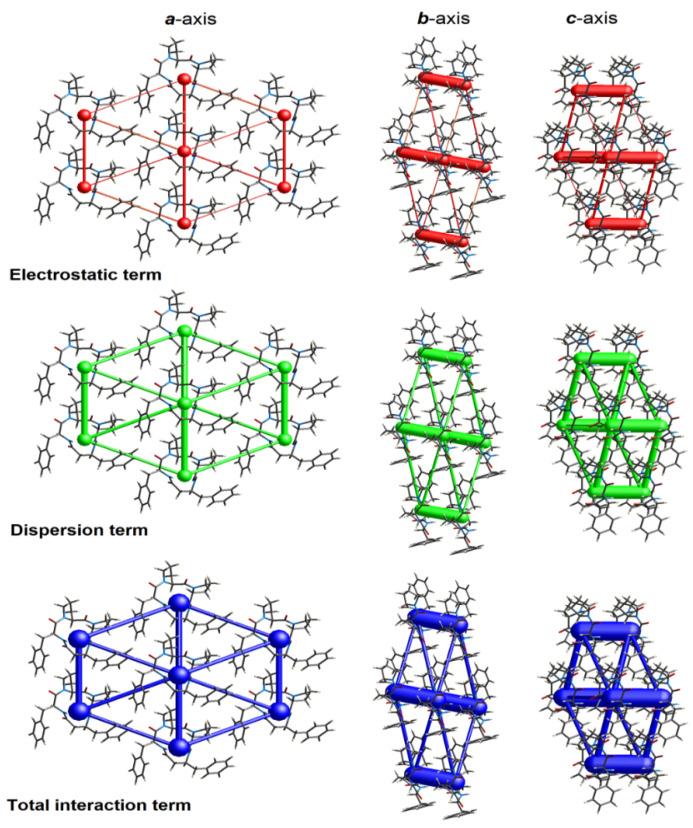
Cylinder-shaped energy frameworks of 4B8M corresponding to the electrostatic and dispersion energy components, and the total energy framework along *a*, *b*, and *c*-axis.

The landscape of 3D topology of intermolecular interactions within crystal 4B8M, termed energy frameworks [93], is visualized in Figure 15, while the molecular interactions towards the reference molecule in a cluster of radius 3.8 Ǻ are shown in Figure 16. By analogy, the corresponding features of YEPVIZ are presented in Figures S9 and S10. The molecular pairs are uniquely color-coded. The width of the cylinders joining the molecules with the size of 150 represents the energy strength between the molecules. According to our results, the dispersion energy term has a greater contribution than the electrostatic term. Thus, weak interactions play the main role in stabilizing the crystal net. The total energy consists of the electrostatic term E′_ele_ = −112.6 kJ/mol, the polarization term E′_pol_ = −39.2 kJ/mol, the dispersion term E′_disp_ = −256.9 kJ/mol, the repulsion term E′_rep_ = 149.1 kJ/mol, and the total energy E′_tot_ = −279.5 kJ/mol, with scale factors of 1.057, 0.740, 0.871, and 0.618, respectively. A similar situation is observed in YEPVIZ. However, the difference between E′_ele_ and E′_disp_ is much greater (E′_ele_ = −19.8 kJ/mol, E′_pol_ = −14.1 kJ/mol, E′_disp_ = −201.3 kJ/mol, E′_rep_ = 73.8 kJ/mol, and E′_tot_ = −161.2 kJ/mol, with scale factors analogical to previous case).

### 2.5. In Silico Pharmacokinetics & Bioactivity Study

Diverse free web platforms for faster screening of pharmacokinetic potential of new compounds, to reduce the time and costs of preliminary research of drug candidates, have been developed in recent years.

To better explore new peptides from the point of view of potential future therapeutic agents as well as further studies on the design and synthesis of derived compounds with better pharmacokinetic profiles, it is necessary to know their bioactivity parameters using free available diverse methodologies as a preliminary inquiries. Prediction of anti-cancer properties has been the point of interest.

First, peptides were pre-estimated for their bioactivity using PeptideRankerScore [94]. Generally, analyzed peptides have very high scores, >0.9, revealing their high bioactivity potential.

**Figure 17:**
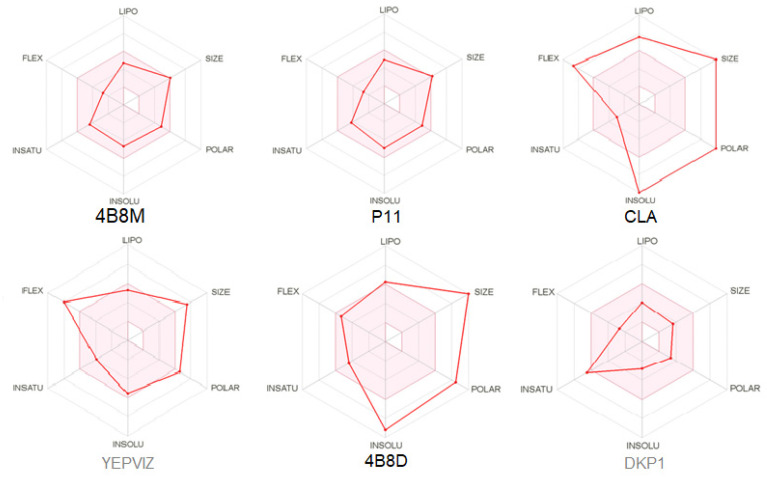
Oral bioavailability graphs for studied peptides. The red zone is suitable for bioavailability. LIPO means lipophilicity, SIZE-molecular weight, POLAR-polarity, INSOLU-insolubility, INSATU-insaturation, FLEX-flexibility.

Bioavailability radars (Figure 17) present tolerated drug-likeness related to physicochemical features [95] for P11, its parent analog 4B8M, and DKP1. BOILED-egg (Brain or IntestinaL EstimateD) diagram, enabling insight into the blood–brain barrier (BBB) permeability and passive human gastrointestinal absorption, is included in Supplementary Materials (Figure S11).

Results of in silico calculations of drug pharmacokinetics and bioactivity, including toxicological properties, related to the ADMET (absorption, distribution, metabolism, excretion, toxicity) [96] are summarized in Tables S11 and S12. Generally speaking, the Lipinski rule of five (the molecular weight ≤ 500, hydrogen bond acceptor (HBA) ≤ 10, hydrogen bond donor (HBD) ≤ 5, lipophilicity (log P) ≤ 5), the measurement of oral bioavailability, should be fulfilled for potential drug molecules [97,98]. Nevertheless, this rule cannot be rigorously applied to peptides because of the inherent molecular weight and number of hydrogen bonds. On the other hand, the bioavailability score can avoid this problem [99]. In this context and this course, 4B8M, P11, and DKP1 pass the Lipinski rule of five (0.55). More specifically, 4B8M, P11, and DKP1 show similar suitability for the Lipinski rule (Pfizer) [97], Veber (GSK) [100], Egan (Pharmacia) [101], and Muegge (Bayer) [102] filters. CLA, 4B8D, and YEPVIZ are suitable for no parameters, including the Ghose (Amgen) [103] filter. 

**Figure 18:**
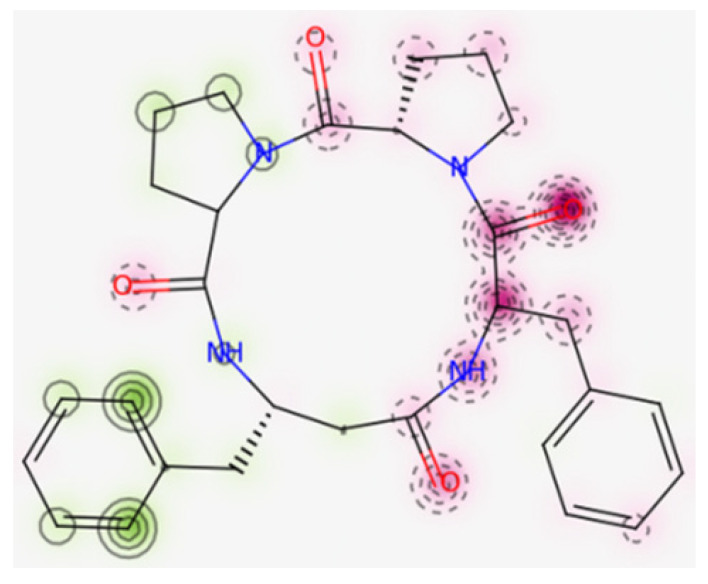
Map of cardiac toxicity of 4B8M obtained from pred-hERG. The increasing number of contour lines, as well as the intensity of the colors, means a greater contribution of atom/fragments of structure to the hERG blockage [105].

Moreover, according to the in silico prediction [104], 4B8M is non-cardiotoxic, while CLA and P11 show potential cardiotoxicity, with 50% confidence values. The probability map of model 4B8M is given in Figure 18, while maps of other peptides are included in Supplementary Materials (Figure S12). Fragments of the structure responsible for positive and negative contributions to the hERG (human ether-a-go-go-related gene) blockage are green and dark pink color, respectively. Gray atoms/fragments mean no contribution [105].

In addition, the full toxicology profile of the compounds analyzed, including probabilities of mutagenicity, carcinogenicity, hepatotoxicity, and estrogenicity, was calculated by the VenomPred web software [106]. Probability values are divided into four ranges: <25%, high confidence in the prediction of a molecule as nontoxic, from 25–50%—low confidence in nontoxicity, from 50–75%—low confidence in toxicity, and >75%—high confidence in toxicity. Cyclo-tetrapeptides show better parameters than their longer analogs, especially in terms of hepatotoxicity (Table S13). 

The prediction of tumor and non-tumor cell line cytotoxicity, using the CLC-pred tool, is based on structure–cell line cytotoxicity relationship by PASS (activity spectra for substances) procedure [107]. Linear peptides show no cytotoxicity for tumor or non-tumor human cell lines. Cyclopeptides reveal cytotoxicity for various tumor cell lines, presented in Table S14. *Pa* values > 0.5 indicate a high probability of action, while *P_i_*—inactivity. 4B8M, P11, CLA, and 4B8D are potentially cytotoxic to breast carcinoma. Furthermore, P11 and 4B8D can be toxic for renal carcinoma, while CLA is cytotoxic against lung carcinoma and central nervous system glioblastoma. 

Considering similarities and differences between cancer types [108,109], these findings can be further exploited in the development of therapeutic agents against melanoma.

**Figure 19:**
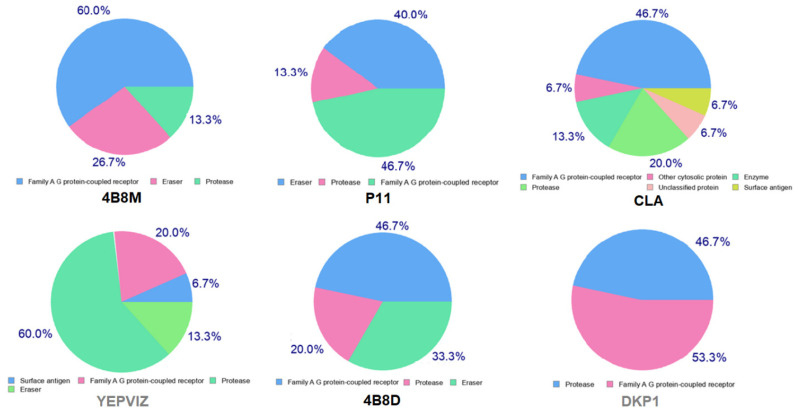
Pie charts showing predicted bio-molecular targets for studied compounds.

Moreover, we estimated the ability of analyzed peptides as potential therapeutical agents via possible interactions with various protein targets, using the SwissTargetPrediction program [110], (Figure 19). Furthermore, bioactivity scores (also known as descriptors of bioactivity), a measure of a molecule’s ability to act with receptors, were predicted via online Molinspiration software [111,112]. They revealed that the studied compounds 4B8M and P11 are active (score is > 0) in relation to the G-protein-coupled receptor (GPCR) and protease inhibitor, while CLA and 4B8D are moderately active (score between −5.0 and 0.0) [113], Table S15. Interestingly, both receptors are associated with melanoma, as well as with many other cancers [114]. 

## 3. Materials and Methods

### 3.1. Chemical Synthesis & Physicochemical Parameters

The synthetic protocol of compounds mentioned below is described in a previous study [115]. All organic reagents as well as CLA were purchased from Sigma-Aldrich Company (St. Louis, MO, USA) and were used as received. In summary, all linear tetrapeptide precursors for cyclic peptides were synthesized by the SPPS method using 2-chlorotrityl- or Wang-resin, except precursors for P0011-6 and P0010-3-3, which were obtained in the solution by 2 + 2 condensation methodology. All cyclization reactions were performed at very high dilution in DCM solution, using HATU/HOAt/2,4,6-collidine. Fmoc amino acid derivatives, resins, and peptide reagents were purchased from Iris Biotech, (Germany). Solvents for peptide synthesis were from JT Baker. 

The molecular weight of model structure 4B8M was calculated for C_29_H_34_N_4_O_4_ = 502.61 g/mol, using a Kratos Kompact Probe MALDI-MS machine (Kratos Analytical Ltd., Manchester, UK). Optical rotation [α]_D_ −113.0^°^ (20 °C, c 0.1 in Acetic acid 99%), HPLC purity > 99.5% [32]. The compound is soluble in organic solvents such as: DMSO, alcohols, and plant oils. The mechanism of action of the cyclic tetrapeptides is described elsewhere [25].

Notably, synthetic analogs of CLA and derived tetracyclopeptides, including 4B8M, have better solubility in water than native CLA [116]. What is more, structural modifications of CLA have four-fold improved solubility in water [25].

### 3.2. Biological Screening

Short peptides were initially dissolved in DMSO, (Sigma-Aldrich, St Louis, MO, USA, #D8418), to obtain final 20 mM stock solutions according to Table S3 and stored at 4 °C until use. 

#### 3.2.1. Generation and Culture of Melanoma Cell Lines

All experiments were performed using patient-derived melanoma cell lines. DMBC28 was obtained from a tumor specimen localized on the back of a 50-year-old man. Melanoma was characterized by Clark level III, Breslow thickness 0.7 mm, mitotic index (MI) 0, and pIIIB stage of disease according to the seventh edition of the American Joint Committee on Cancer (AJCC) for melanoma. DMBC29 was obtained from a tumor specimen localized on the face of a 61-year-old woman. Melanoma was characterized as nodular melanoma (NM) by Clark level III, Breslow thickness 3.5 mm, mitotic index (MI) 2, and pIIIC stage of disease according to the seventh edition of the AJCC for melanoma. The study was approved by the Ethical Commission of the Medical University of Lodz (identification code: RNN/84/09/KE) and informed consent was obtained from the patients. 

**Figure 20:**
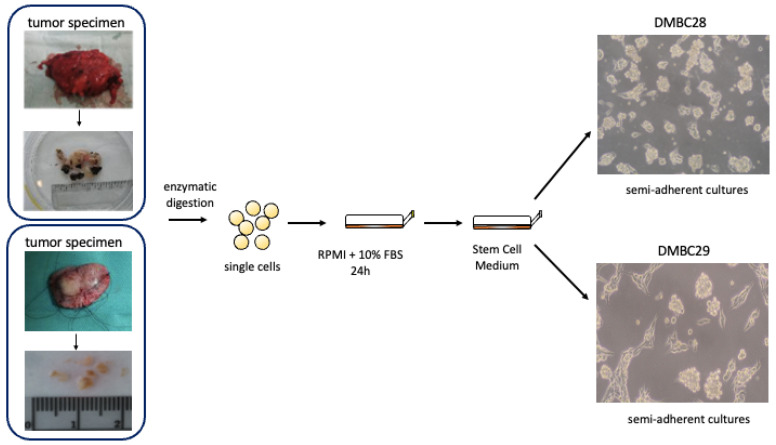
Simplified scheme of DMBC28 and DMBC29 isolation.

Surgical specimens, after several washes were sliced with scissors and incubated in Hanks’ Balanced Salt Solution (HBSS; Sigma-Aldrich, St Louis, MO, USA) supplemented with 3 mM calcium chloride and 1 mg/mL collagenase IV for 2–3 h at 37 °C. After the collagenase digestion, DNase I (10 μg/mL) was added, and cells were filtered through a 70 μm pore size filter. Cells were cultured in complete medium (RPMI-1640 with 10% FBS) for 24 h to remove dead and nonadherent cells, then they were transferred to serum-free stem cell medium (SCM), consisting of Dulbecco’s Modified Eagle’s Medium (DMEM/F12) low osmolality medium (Lonza, Basel, Switzerland) supplemented with: B-27 supplement (Gibco, Paisley, UK), 10 ng/mL basic fibroblast growth factor (bFGF) (Corning, Corning, NY, USA), 20 ng/mL epidermal growth factor (EGF) (Corning, Corning, NY, USA), insulin (10 µg/mL) (Sigma-Aldrich, St Louis, MO, USA), heparin (1 ng/mL) (Sigma-Aldrich, St Louis, MO, USA), 100 IU/mL penicillin, 100 µg/mL streptomycin, and 2 µg/mL fungizone B. Cell cultures were maintained in low-adherent flasks (NUNC) at 37 º C in a humidified atmosphere containing 5% CO_2_ and the medium was exchanged twice a week [117]. DMBC28 and DMBC29 cells represent ‘semi-adherent’ cultures, in which both floating and adherent cells are observed. The simplified scheme of melanoma cell isolation and morphology of DMBC28 and DMBC29 cells is presented below (Figure 20).

#### 3.2.2. APA Assay

To assess the viable cell number, the activity of acid phosphatase was measured colorimetrically. Briefly, melanoma cells were plated at a density of 3.2 × 10^3^ viable cells per well in 96-well plates, 2 h before short peptide treatment. Then, cells were exposed to short peptides (0.1 μM;1 μM; 10 μM; 20 μM; 50 μM) or a vehicle (0.1%; 0.25% DMSO) and cells were grown for 24, 48, and 72 h. After indicated time intervals, the plates were centrifuged, and the medium was replaced with 100 µL of assay buffer containing 0.1 mol/L sodium acetate (pH = 5), 0.1% Triton X-100, and 5 mmol/L p-nitrophenyl phosphate (Sigma-Aldrich, St Louis, MO, USA). The plates were incubated for 2 h at 37 °C and the reaction was stopped by adding 10 µL of 1 mol/L NaOH. The absorbance values were measured at 405 nm using a microplate reader Infinite M200Pro (Tecan, Salzburg, Austria). 

#### 3.2.3. Time-Lapse Microscopy (IncuCyte ZOOM)

A time-lapse fluorescence microscope system (IncuCyte, Essen Bioscience) was used to detect real-time changes in cell confluence during short peptide treatment. Briefly, melanoma cells were plated at a density of 8 × 10^3^ viable cells per well in 96-well plates 2 h before short peptide treatment. Then, cells were exposed to short peptides (10 μM; 20 μM; 50 μM) or vehicle (0.1%; 0.25% DMSO) and monitored every 4 h. The changes in the area occupied by melanoma cells in control, vehicle-treated (0.1%; 0.25% DMSO), and short peptide-treated cultures over time were assessed fold-change relative to the confluence at time T_0_. Data were analyzed using the IncuCyte Zoom original software. 

#### 3.2.4. Flow Cytometry

Flow cytometry and propidium iodide (PI) staining (Sigma-Aldrich, St Louis, MO, USA #81845) was used to assess changes in the percentages of viable and dead cells. Melanoma cells were plated at a density of 7 × 10^4^ viable cells per well in 24-well plates and incubated for 2 h before treatment. Then, cells were exposed to 4B8M-12, P11, P11L, and CLA (0.1 μM;1 μM; 10 μM; 20 μM; 50 μM) or a vehicle (0.1%; 0.25% DMSO). After 48 h cell suspension from each well was taken to assess relative changes in the percentages of viable and dead cells (FACSVerse flow cytometer and FACSuite software; Becton Dickinson, San Jose, CA, USA). 

#### 3.2.5. Statistical Analysis

All statistical calculations were performed with GraphPad Prism 9 software. Student’s *t*-test was used to determine significant differences between the mean values of the tested parameters. The differences were considered significant at *p* < 0.05.

### 3.3. Single-Crystal X-ray Diffraction (SC-XRD)

Crystallization of suitable crystals for single-crystal X-ray analysis was not an easy task due to the tendency of 4B8M to form syrup-like amorphous phase or ‘twinned’ crystals. Finally, after many attempts, acceptable quality single crystals were grown via slow evaporation of a glacial acetic acid.

X-ray data were collected on a Rigaku OD XtaLAB Synergy, Dualflex, Pilatus diffractometer using graphite monochromated Cu*K*α radiation (*λ* = 1.54158 Å) at a temperature of 100 K, using Crysalis, structure determination and refinement by Olex2 [118]. The structure was solved with the XT [119] structure solution program using intrinsic phasing and refined with the XL [120] refinement package using least-squares minimization. A CIF file containing complete information of the studied structure was deposited with CCDC, deposition number 2158219 is freely available upon request from the Director, CCDC, 12 Union Road, Cambridge CB2 1EZ, UK (Fax: + 44-1223-336033; e-mail: deposit@ccdc.cam.ac.uk).

All molecular interactions in the crystals of 4B8M and YEPVIZ were identified by the PLATON [121] and Mercury [116] programs.

### 3.4. Computational Methods

#### 3.4.1. Density Functional Theory (DFT) Calculations

The geometry of neutral 4B8M and YEPVIZ molecules in ground singlet spin states obtained from X-ray analysis of single crystals [39] was completely optimized using the Gaussian 09 program [122]. For the calculations of stable conformers in both the gas phase and the solvated state, the density functional theory [123,124,125] with the hybrid functional B3LYP [126,127,128] and the polarized triple-ζ 6–311++G(d,p) basis sets from the Gaussian library [122] were used. The conductor-like polarizable continuum model (CPCM) [129,130,131] was used to evaluate the effect of hydration on the structure of the 4B8M and YEPVIZ conformers studied. The stability of the optimized geometries was checked by vibrational analysis (no imaginary vibrations). Atomic charges were evaluated in terms of natural bond orbital (NBO) analysis [132,133,134,135,136]. The gas-phase and solvated-state molecular structures obtained from theoretical calculations were discussed and compared with the X-ray structure of those compounds in the crystalline state.

#### 3.4.2. Hirshfeld Surface Calculations

The Hirshfeld surface (HS) studies, including 3D HS maps, 2D fingerprint plots (FP), molecular electrostatic potential (EP), and energy frameworks (EF) [92,93,137], were performed using CrystalExplorer 17 software [138], and a final refined crystallographic information file (CIF) from single-crystal X-ray analysis as the input. HS maps were generated by d_norm_ (normalized sum of d_e_ and d_i,_ in terms of the van der Waals radii), d_i_ (distance from HS to the nearest internal atom to the surface), d_e_ (distance from HS to the nearest external atom to the surface), shape index (a function that depends on the HS flatness/curvature), curvedness (a function that depends on the HS concavity/convexity), fragment patch (coordination environment of the molecule in crystal) [93].

The EP surface map was calculated using wave function STO-3-G basis sets at the Hartree–Fock theory level over the range of −0.025 au. 

The EF was constructed by single-point molecular wave functions at B3LYP/6–31 G(d,p) level of theory, within a cluster of the radius of 3.8 Ǻ [93,138,139], on the basis of the following equation:

E_tot_ = k_ele_ E′_ele_ + k_pol_E′_pol_ + k_dis_ E′_dis_ + k_rep_E′_rep_

where k characterizes scale factor values, E′_ele_—the electrostatic energy, E′_pol_—the polarization energy, E′_dis_—the dispersion energy, and E′_rep_—the exchange repulsion energy. 

The enrichment ratios (ER) were calculated on the basis of interactions between various chemical species (x, y) and proportions derived from the HS content, using the below equations [140].

Sx = Cxx + ½∑_(y≠x)_Cxy; Rxy = 2SxSy; Exy = Cxy/Rxy

where Cxy is a contribution of a corresponding atomic contact to the HS; Exy is the enrichment ratio. 

#### 3.4.3. In Silico Analysis

The ADMET profile was estimated through the Swiss ADME tool from the Swiss Institute of Bioinformatics [96]. 

Additional data were calculated using the pkCSM platform [108].

Full toxicology profiles of analyzed compounds, including probabilities of mutagenicity, carcinogenicity, hepatotoxicity, and estrogenicity were calculated by the VenomPred web tool [106].

The cardiac toxicity of peptides analyzed, for the early prediction of potential hERG blockers and nonblockers, was calculated by the pred-hERG 4.2 web tool, freely accessed in February 2022 [105].

The bioactivity scores were calculated by the PeptideRankerScore tool (provider: University College Dublin, Ireland [94].

Molecular target probabilities, essential to predict the phenotypical side effects or possible cross-reactivity related to the action of analyzed peptides, were calculated through the free online tool.

The predictions of tumor and non-tumor cell line cytotoxicity were performed using the CLC-pred tool, which is based on structure–cell line cytotoxicity relationships by PASS (activity spectra for substances) procedure [107]. 

All ‘in silicon’ simulations were carried out in February 2022. 

The structures of peptides were converted into canonical simplified molecular input line entry specification (SMILES). 

## 4. Conclusions and Future Perspectives

In summary, in this study, we applied a comprehensive approach to analyze peptides containing the (Pro-Pro-Phe-Phe-) scaffold and its analogs with synthetic amino acids as potential drug lead agents. 

All compounds were previously tested for their likelihood to be highly bioactive. We found that selected cyclic peptides, contrary to their linear analogs, showed promising anti-cancer activity. Cyclo(Leu-Ile-Ile-Leu-Val-Pro-Pro-Phe-Phe-), denoted as CLA, presented cytotoxic, while cyclo(Pro-homoPro-β^3^^-^homoPhe-Phe-), P11,—exerted cytostatic effects against melanoma, one of the most fatal cancers. The study was performed on patient-derived melanoma cells that were cultured in a stem cell medium. It has been found that P11 reduced the viable cell number by inhibiting cell proliferation without inducing cell death. CLA in turn exerted a cytotoxic effect on melanoma cells since the reduction in viable cell number was accompanied by massive cell death as assessed by flow cytometry after staining with propidium iodide that penetrates dead or damaged cells, and by microscopy. Of note, CLA was the most effective as it exerted its strong anti-melanoma activity at 20 µM, whereas the cytostatic effect of P11 was mild and delayed at this concentration. Cytostatic effects of P11 could be enhanced by using a higher concentration (50 μM). P11L, the linear variant of P11, did not affect melanoma cell proliferation and viability. Thus, our study confirmed that short cyclic peptides exert better biological activity compared to their linear counterparts. This can be explained by their conformational rigidity and higher stability. While we did not perform a cell permeability assay at this stage of the study, we can assume based on the observed cytostatic and cytotoxic effects of cyclic peptides that their higher activity compared to linear ones can be also explained by improved cell membrane permeability.

In silico predictions were helpful for a preliminary bio-pharmacologic insight into the relevance of homoPro and β^3^homoPhe substitutions. Tetracyclopeptides, such as P11 and its parent analog cyclo(Pro-Pro-β^3^homoPhe-Phe-), called 4B8M, had a better framework than CLA. All compounds showed a lack of mutagenicity. In turn, 4B8M was the only non-cardiotoxic compound. All studied cyclo-peptides revealed significant anticancer activity in CLC-Pred and PASS web tools. 4B8M, P11, and CLA exhibited cytotoxicity for tumor human cell lines related to breast carcinoma. In addition, P11 was cytotoxic for renal cancer, while CLA—lung carcinoma and glioblastoma. 

The first model structure in this class of compounds, 4B8M, crystallized in triclinic space group P1, with one molecule in the crystal lattice’s asymmetric unit. The DFT studies revealed that geometry differences between X-ray and DFT optimized structures in a vacuum and aqueous solutions can be explained by intermolecular hydrogen bonds and crystal-packing forces of both compounds. Frontier molecular orbitals of 4B8M are located prevailingly at phenyl rings unlike the unoccupied molecular orbital of YEPVIZ, the most similar structure deposited in the CSD, located at its tetrapeptide ring. The corresponding orbital energies indicate the high electron-acceptor ability and stability of both compounds. The results on energy frameworks revealed that the dispersion forces related to weak intercontacts are relevant to the topology of the interaction. C-H^…^O, C-H^…^π, and C-O^…^π stabilize supramolecular architecture. The potential of rigid cyclotetrapeptides containing β^3^homo cyclic amino acids to form nanotubular self-organization cannot be ignored. It is an important note for the development of modern strategies related to effective theranostics and drug delivery in oncology. 

In summary, this report represents progress contributing to expanding knowledge on preferred bioactivity and reactivity of cyclo(Pro-Pro-Phe-Phe-) sequence and its modifications in terms of incorporation of synthetic amino acids. These promising findings can serve as a starting point to the docking studies to understand the modes of action of these cyclo-peptides. Such studies could supplement our hitherto observations on the immunological mechanisms of action, as in the case of 4B8M. It is worth noting that they were predicted to bind to G protein-coupled receptors and protease inhibitors. Short cyclo-peptides containing β^3^homoPro and β^3^homoPhe amino acids have the potential for the treatment of melanoma. Nevertheless, more advanced experimental studies are needed. It is reasonable to suppose that this first structural consideration on the cyclo(Pro-Pro-Phe-Phe) scaffold can be a stepping stone for further endeavors on its modifications providing direction to the synthesis of safe and effective anti-cancer drugs, delivery systems, and diagnostics, especially against highly drug-refractory melanoma. 

## Figures and Tables

**Table 3:** Crystallographic data for 4B8M.

Formula	C_29_H_34_N_4_O_4_
Molecular weight	502.60
Temperature (K)	100
Crystal system, space group	triclinic, P1
a, b, c (Å); α, β, ɣ (^°^)	5.6034 (2), 9.9007 (3), 12.5815 (4); 67.2640 (10), 87.2840 (10), 77.831 (2)
V (Å^3^)	628.84(4)
*Z*	1
Radiation type	CuKα
µ (mm^−1^)	0.722
No. of reflections	7452
No. of unique reflections	4444/3656
No. of parameters	343
No. of restraints	3
R_int_	0.0130
T_min_, T_max_	0.878, 0.897
R[F^2^ > 2σ(F^2^)], wR(F^2^), S	0.0293, 0.0783, 0.994
CCDC number	2158219

**Table 4:** Hydrogen bond geometry for 4B8M (Å, °).

D-H^…^A	D-H	H^…^A	D^…^A	D-H^…^A
* N3-H3N^…^N2	0.85 (3)	2.39 (3)	2.783 (3)	109 (2)
C3-H3A^…^O4 ^i^	0.97	2.53	3.467 (3)	163
* C29-H23^…^O3	0.98	2.34	2.785 (3)	107
C9-H9^…^O1 ^i^	0.98	2.34	3.172 (3)	143
C19-H19A^…^O2 ^ii^	0.97	2.33	3.139 (4)	140
C16-H16^…^O4 ^iii^	0.93	2.46	3.379 (3)	169
* C11-H11^…^O2	0.98	2.41	2.830 (4)	105

* Intramolecular H-bonds. Symmetry codes: (^i^) −1 + x, y, z; (^ii^) 1 + x, y, z; (^iii^) −1 + x, y, −1 + z.

## Data Availability

Not applicable.

## Supplementary Materials

The following are available online at https://www.mdpi.com/article/10.3390/ijms23137173/s1.

## Abbreviations

ADMET	Absorption: distribution, metabolism, excretion, toxicity
APA	Acid phosphatase activity
BOILED	Brain or intestinal estimated
BBB	Blood–brain barrier
CNS	Central nervous system
CLA	Cyclolinopeptide A
CLC	Cell line cytotoxicity
CPCM	Conductor-like polarizable continuum model
CSD	Cambridge Structure Database
CYP	Cytochrome P450 enzymes
2D	Two dimensionality
3D	Three dimensionality
DFT	Density functional theory
DMSO	Dimethyl sulfoxide
de	Distance from HS to the nearest external atom to the surface
di	Distance from HS to the nearest internal atom to the surface
dnorm	Normalized distance
E′_dis_	Dispersion energy term
E′_ele_	Electrostatic energy term
E′_pol_	Polarization energy term
E′_rep_	Repulsion energy term
E′_tot_	Total energy
EMA	European Medicines Agency
ER	Enrichment ratio
EP	Electrostatic potential
FLEX	Flexibility
FP	Fingerprint plot
GI absorption	Gastrointestinal absorption
HBA	Hydrogen bond acceptor
HBD	Hydrogen bond donor
HBSS	Hanks’ balanced salt solution
hERG	Human ether-a-go-go-related gene
HOMO	Highest occupied molecular orbital
HS	Hirshfeld surface
Ile	Isoleucine
INSATU	Insaturation
INSOLU	Insolubility
LD50	Lethal dosage
Leu	Leucine
LIPO	Lipophilicity
LSAM	Long-range Aufbau module
LUMO	Lowest unoccupied molecular orbital
NBO	Natural bond orbital
OCT2	Organic cation transporter 2
Pa	Probability of action
Pi	Probability of inactivity
PAINS	Pan-assay interference
POLAR	Polarity
Pro	Proline
RCSB PDB	Research Collaboratory for Structural Bioinformatics Protein Data Bank
SC-XRD	Single crystal X-ray diffraction
SIZE	Molecular weight
SMILE	Simplified molecular input line entry specification
TPSA	Topological polar surface area
Val	Valine
WHO	World Health Organization
