# Supplementary material for: Structural and Biofunctional Insights into the Cyclo(Pro-Pro-Phe-Phe-) Scaffold from Experimental and In Silico Studies: Melanoma and Beyond
Source: Int J Mol Sci. 2022 Jun 28;23(13):7173. doi: 10.3390/ijms23137173 (PMC9266943; doi:10.3390/ijms23137173)
Supplement: Supplementary file 1 [file ijms-23-07173-s001.zip › ijms-1700729-supplementary.pdf]

Structural and Biofunctional Insights into the Cyclo(Pro-Pro-Phe-Phe-) Scaffold from Experimental and In Silico Studies: Melanoma and Beyond

Joanna Bojarska[1*], Martin Breza[2], Milan Remko[3], Małgorzata Czyż[4], Anna Gajos-Michniewicz[4], Michał Zimecki[5], Krzysztof Kaczmarek[6], Izabela D. Madura[7], Jakub M. Wojciechowski[8], and Wojciech M. Wolf[1]

[1] Institute of Ecological and Inorganic Chemistry, Faculty of Chemistry, Lodz University of Technology, Żeromskiego 116, 90-924 Lodz, Poland
[2] Department of Physical Chemistry, Slovak Technical University, Radlinskeho 9, SK-81237 Bratislava, Slovakia
[3] Remedika, Luzna 9, SK-85104 Bratislava, Slovakia
[4] Department of Molecular Biology of Cancer, Medical University of Lodz, Poland
[5] Institute of Immunology and Experimental Therapy, Polish Academy of Sciences, ul. Weigla 12, 53-112 Wrocław, Poland
[6] Institute of Organic Chemistry, Faculty of Chemistry, Lodz University of Technology, Żeromskiego 116, 90-924 Lodz, Poland
[7] Warsaw University of Technology, Faculty of Chemistry, Poland
[8] Rigaku Europe SE-Hugenottenallee 167, 63263, Neu-Isenburg, Germany

* Correspondence: joanna.bojarska@p.lodz.pl

Supplementary Materials

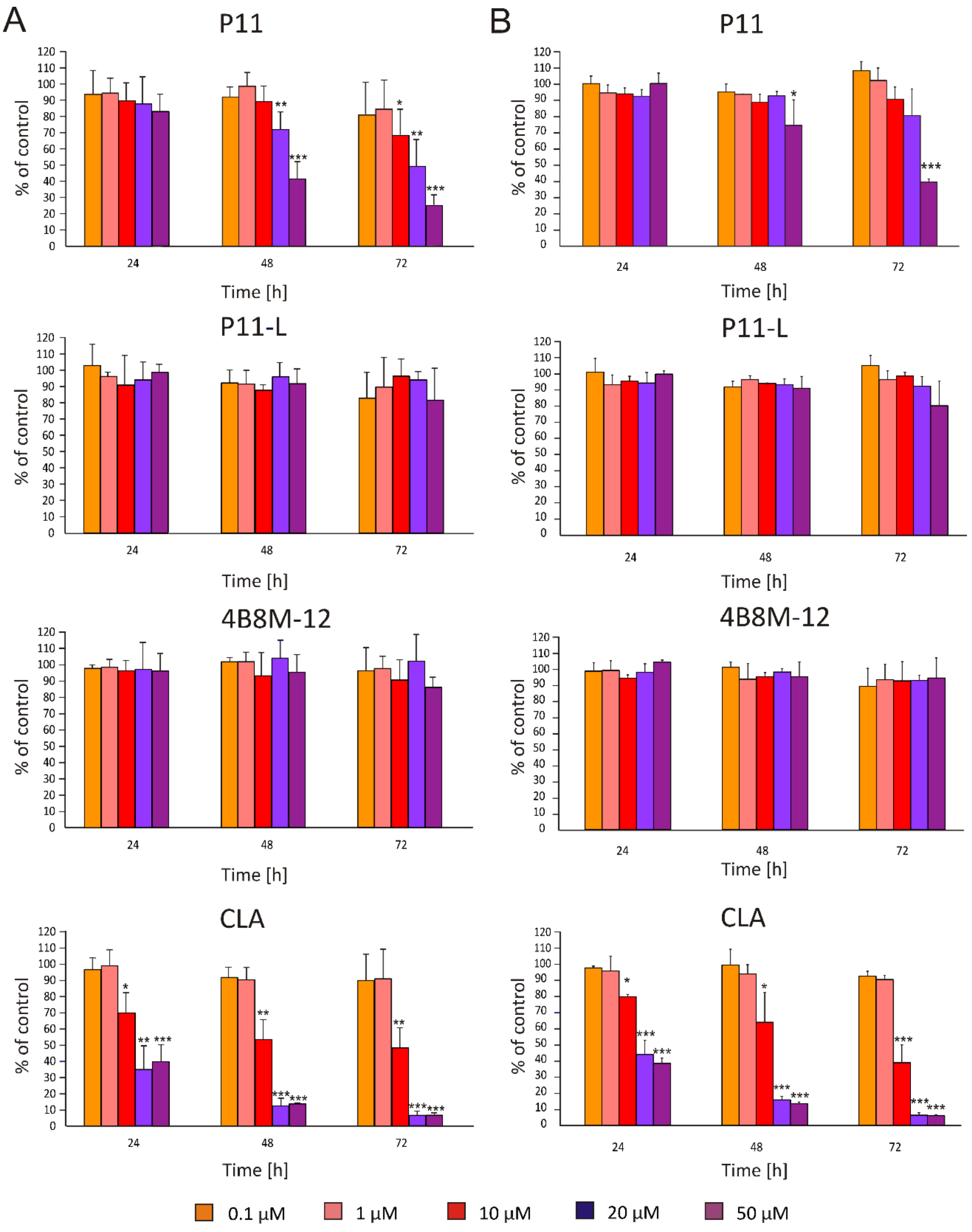

Figure S1. Viability of DMBC29 cells (panel A) and DMBC28 cells (panel B) treated with short peptides assessed in APA assay and expressed as % of control. Bars represent mean values of three biological replicates ± SD. Statistically significant differences are indicated as * $p < 0.05$; ** $p < 0.005$; *** $p < 0.001$.

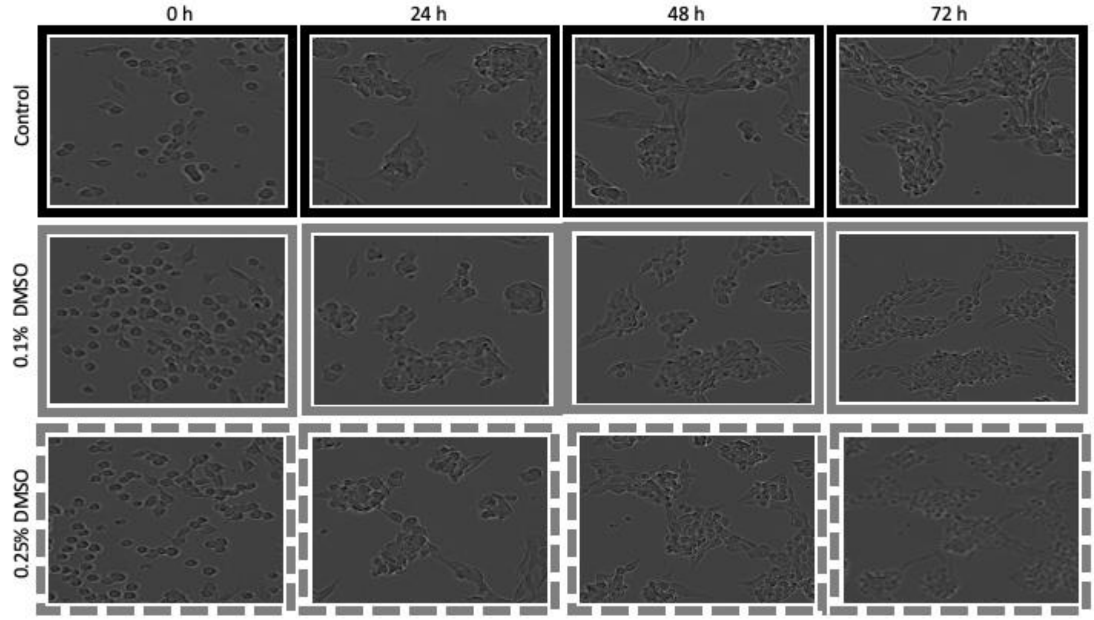
0 h
24 h
48 h
72 h
Control
0.1% DMSO
0.25% DMSO

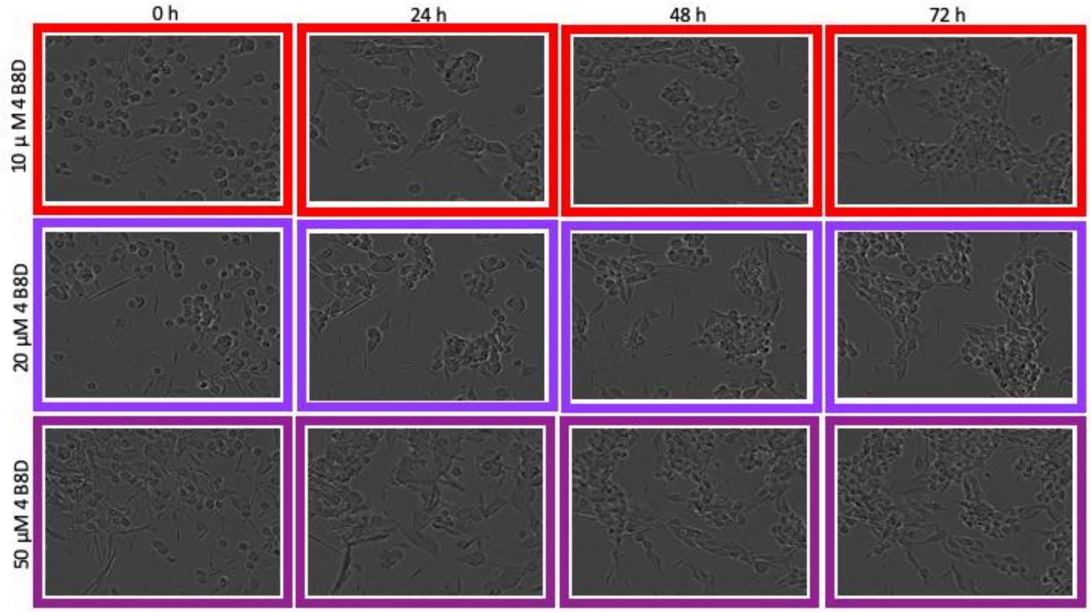
0 h
24 h
48 h
72 h
10 µ M 4 B8D
20 µM 4 B8D
50 µM 4 B8D

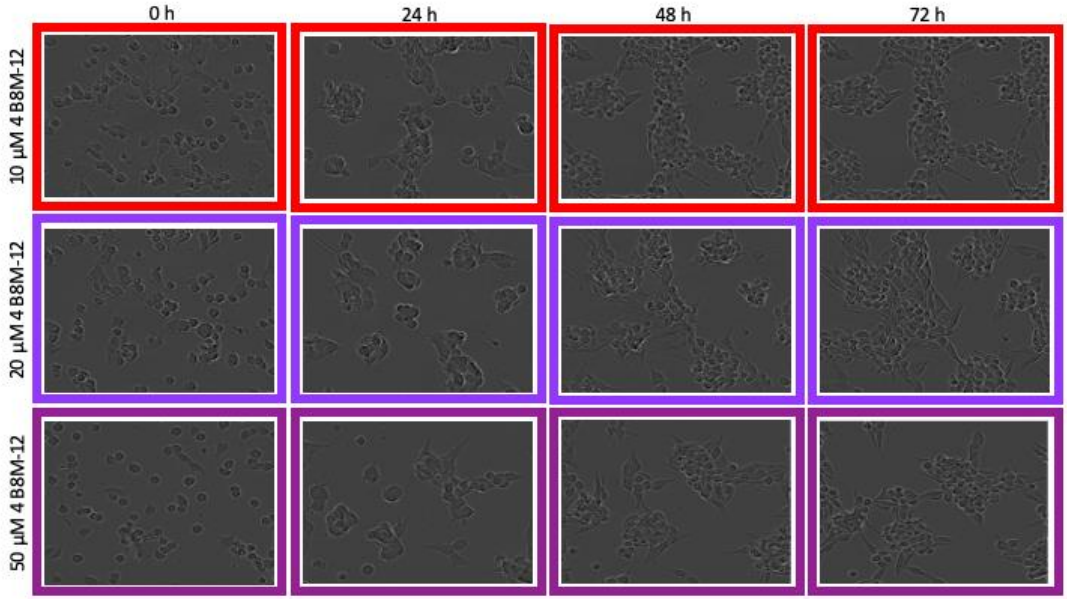
0 h
24 h
48 h
72 h
10 µM 4 B8M-12
20 µM 4 B8M-12
50 µM 4 B8M-12

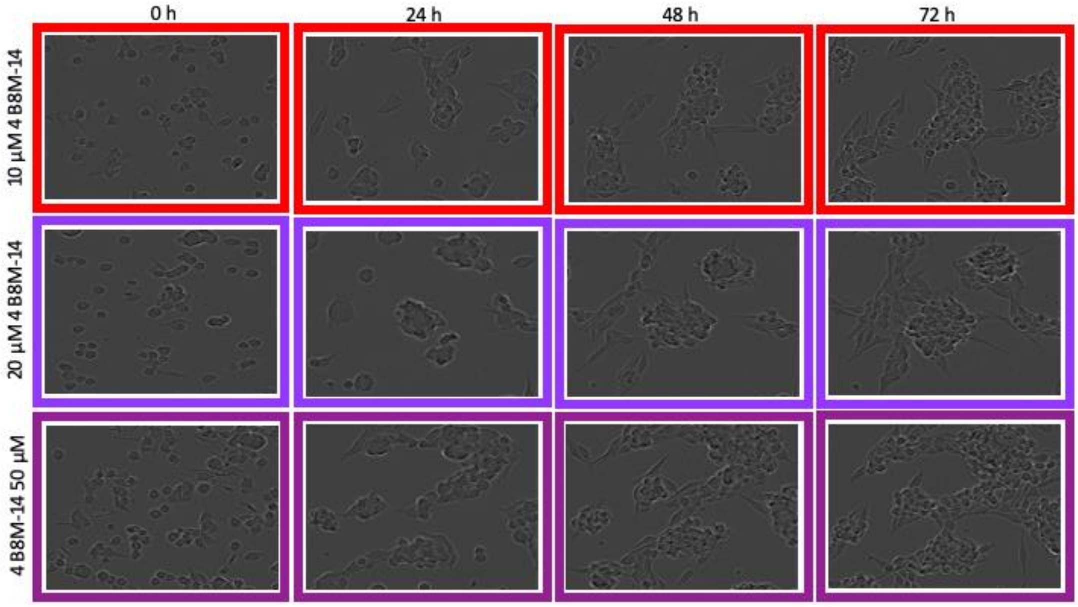
0 h
24 h
48 h
72 h
10 µM 4 B8M-14
20 µM 4 B8M-14
4 B8M-14 50 µM

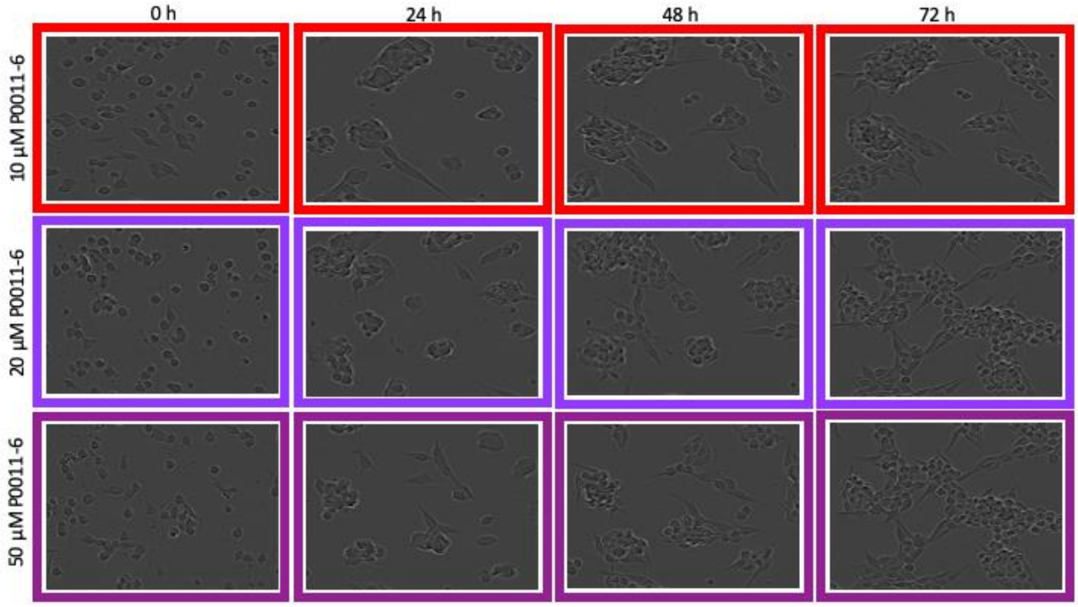
0 h
24 h
48 h
72 h
10 µM P0011-6
20 µM P0011-6
50 µM P0011-6

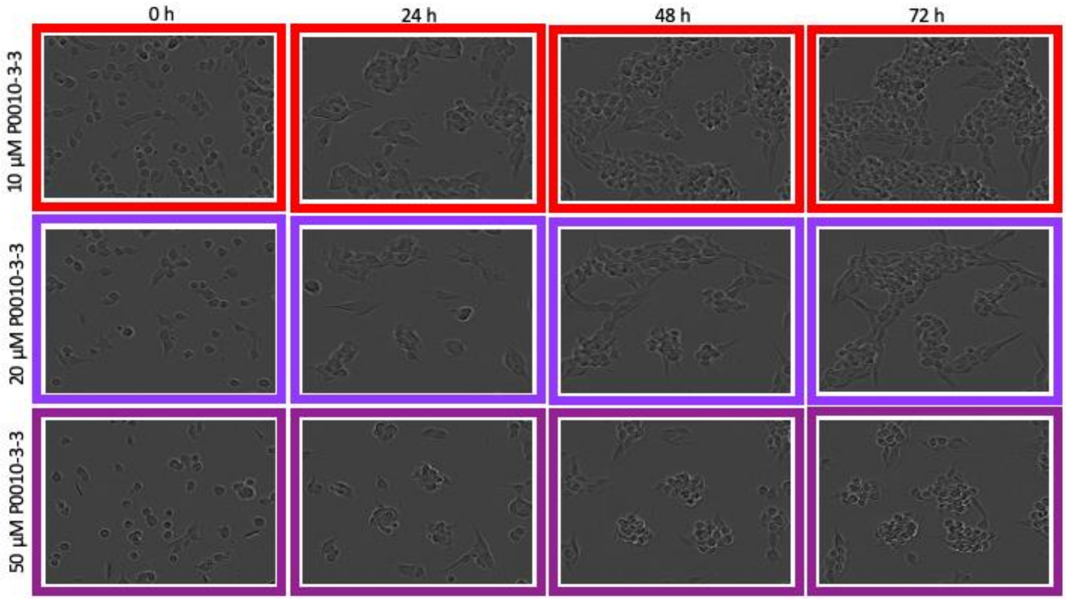
0 h
24 h
48 h
72 h
10 µM P0010-3-3
20 µM P0010-3-3
50 µM P0010-3-3

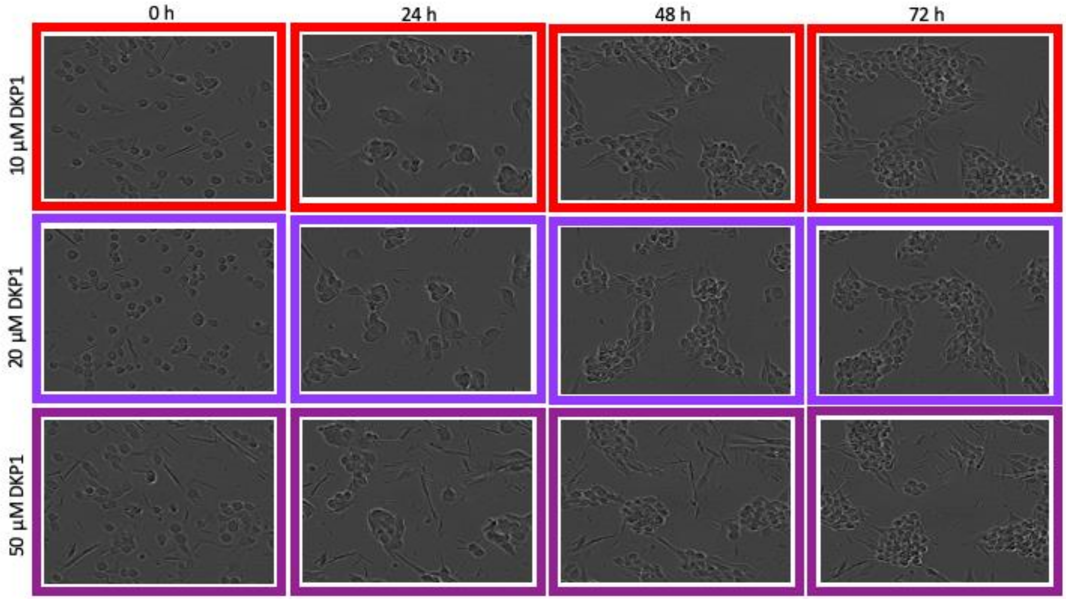
0 h
24 h
48 h
72 h
10 µM DKP1
20 µM DKP1
50 µM DKP1

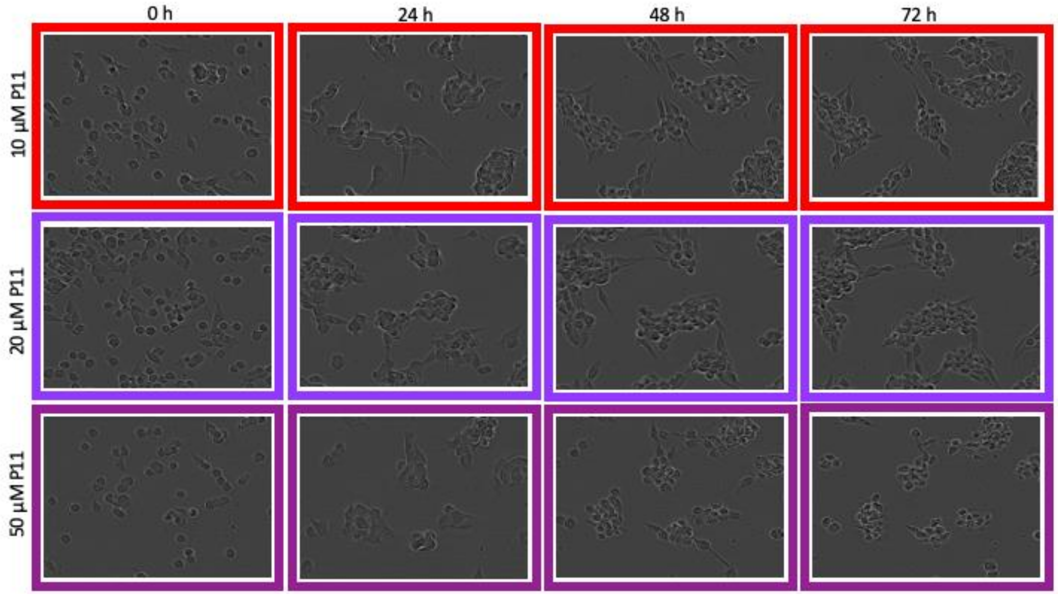
0 h
24 h
48 h
72 h
10 µM P11
20 µM P11
50 µM P11

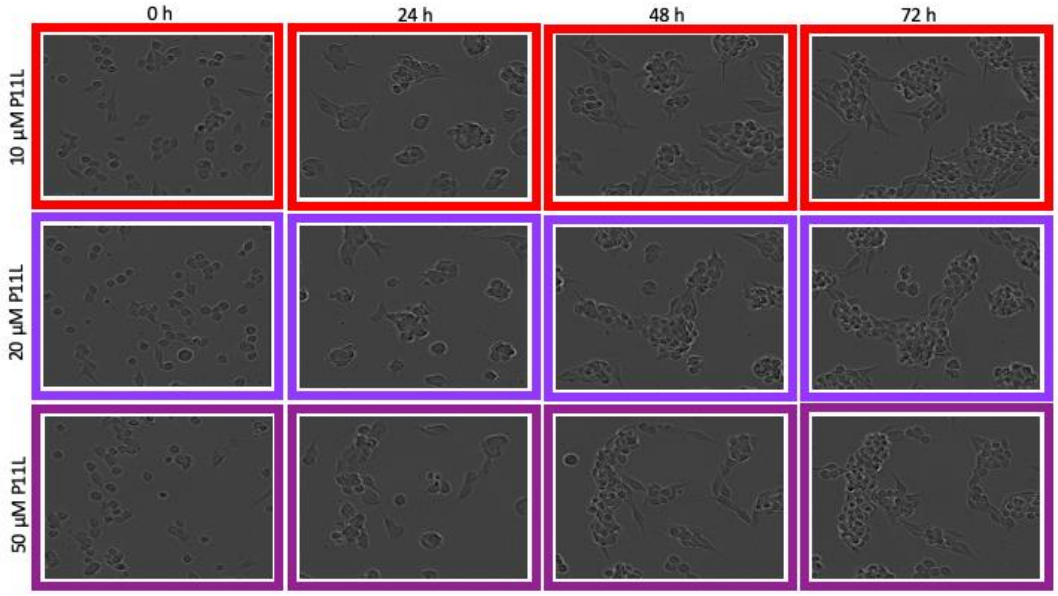
0 h
24 h
48 h
72 h
10 µM P11L
20 µM P11L
50 µM P11L

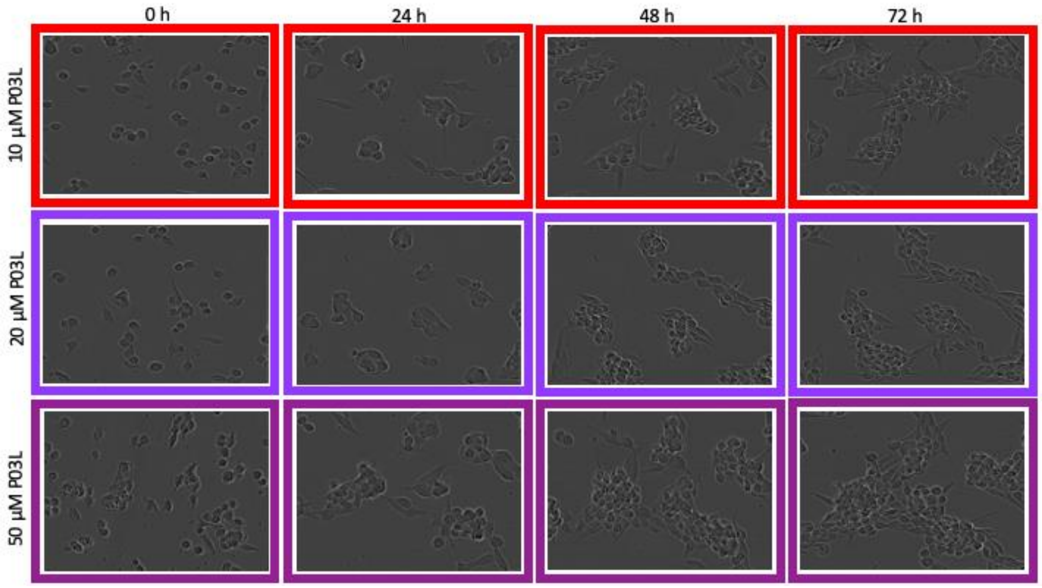
0 h
24 h
48 h
72 h
10 µM P03L
20 µM P03L
50 µM P03L

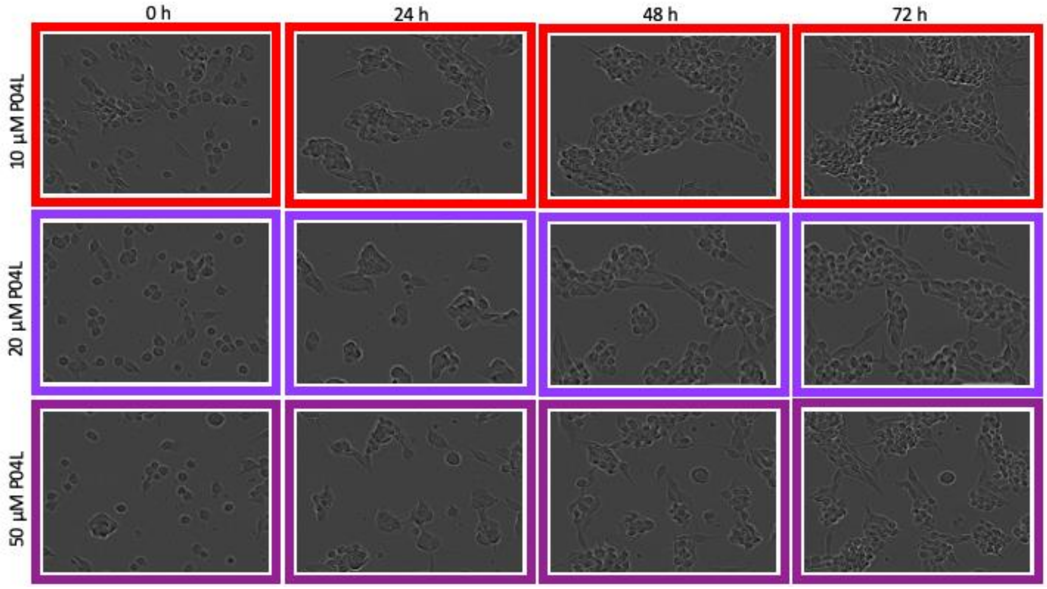
0 h
24 h
48 h
72 h
10 µM P04L
20 µM P04L
50 µM P04L

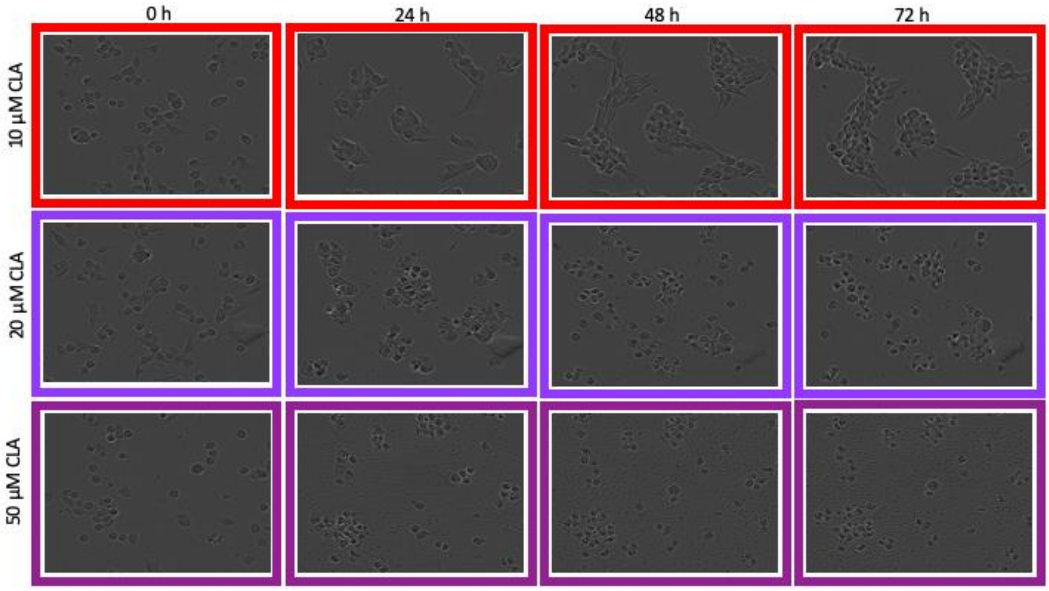

Figure S2. DMBC29 cells were exposed to a vehicle (0.1%; 0.25% DMSO) or short peptides at different concentrations (color framed) for 24, 48 and 72 h. The microphotographs were taken under the microscope in IncuCyte.

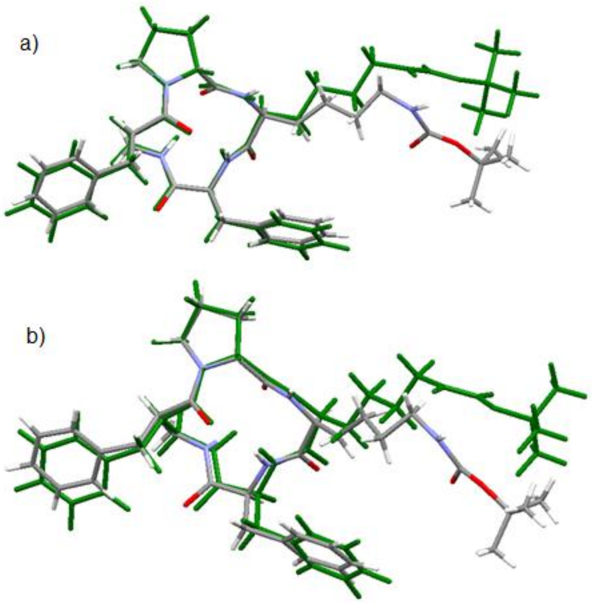

Figure S3. Superposition of the the X-ray structure of X-ray_structure of JOC ArvidssonYEPVIZ and the B3LYP optimized YEPVIZ in the gas phase (a) and B3LYP optimized solvated YEPVIZ (b). The optimized structures are in green.

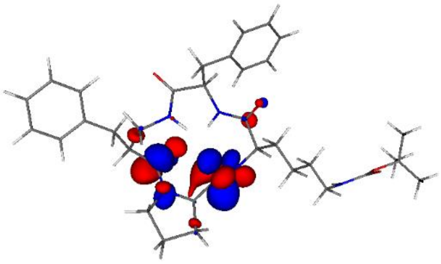

LUMO

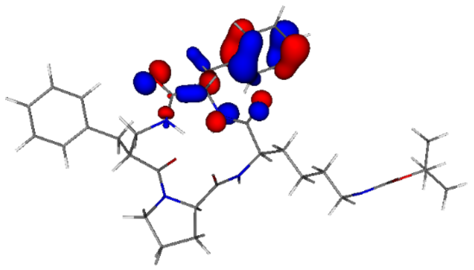

HOMO

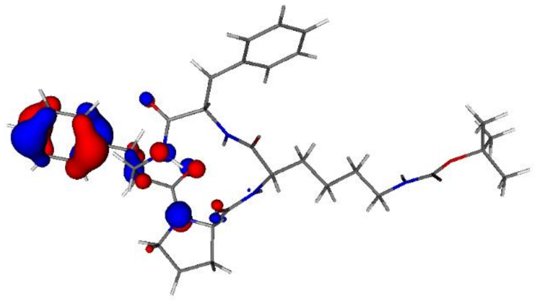

HOMO - 1

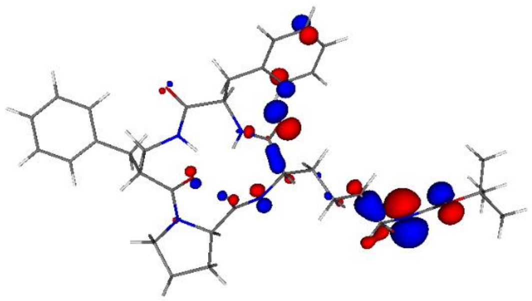

HOMO – 2

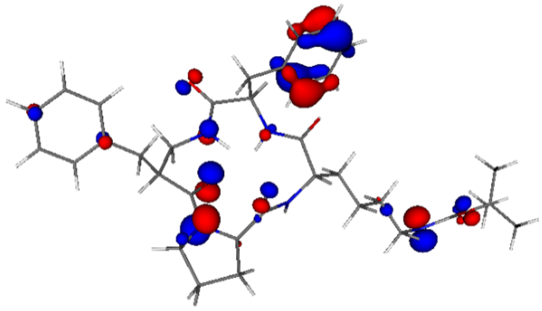

HOMO - 3

Figure S4. Frontier MOs of YEPVIZ (0.05 a.u. isosurface).

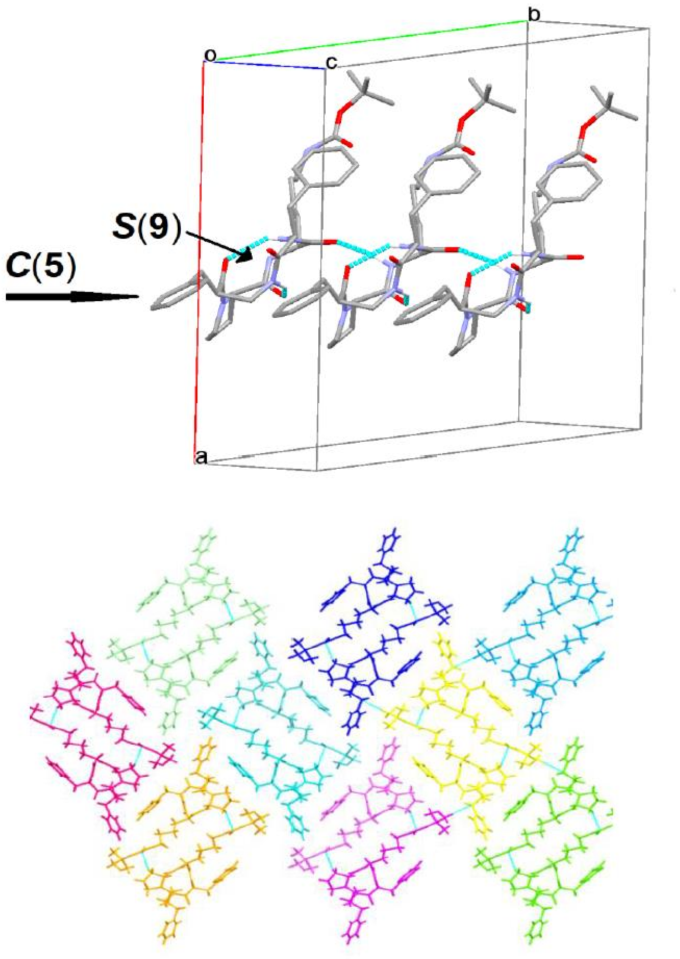

Figure S5. YEPVIZ – pseudohexaonal packin of 1D lader-like LSAMs. Each LSAM has a different colouring.

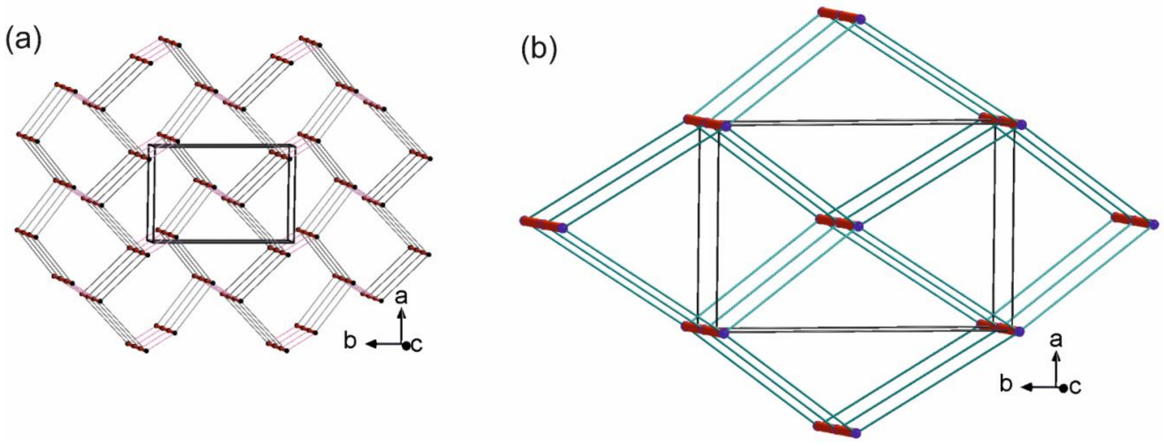

Figure S6. a) Simplified packing in YEPVIZ. Molecules are reduced to their cenrte of gravity, solid red lines show the strongest hydrogen bonds while thin pink and gray are in line with C-H···O and C-H···◎ interactions, respectively. b) Blue dots (nodes) represent the centere of gravity of a dimer formed by weak C-H···O interactions, i.e. a LSAMs. Lines show the connections *via* weak interactions. The topology of this network is similar to pcu.

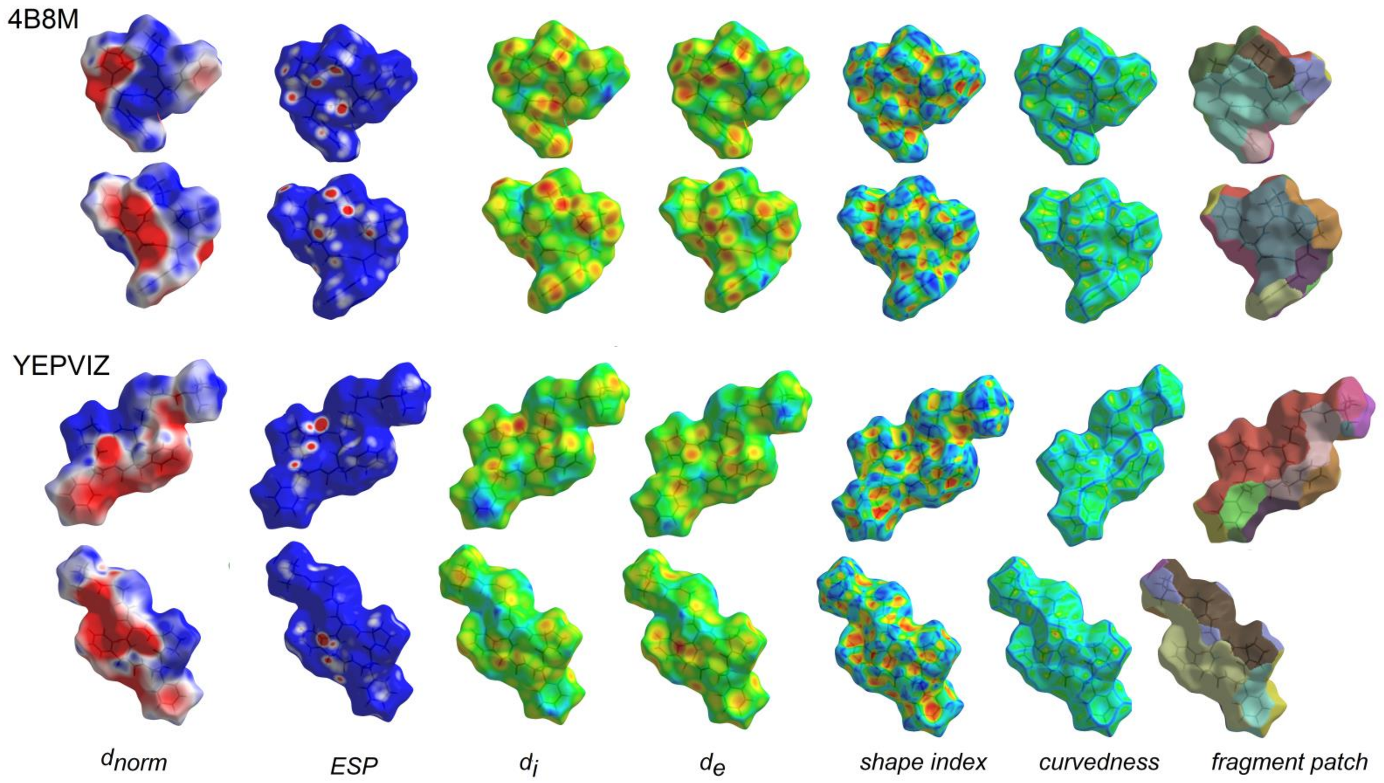

Figure S7. View of the 3D Hirshfeld surface of 4B8M and YEPVIZ mapped with d_{norm}, d_i, d_e, shape index, curvedness and fragment patch.

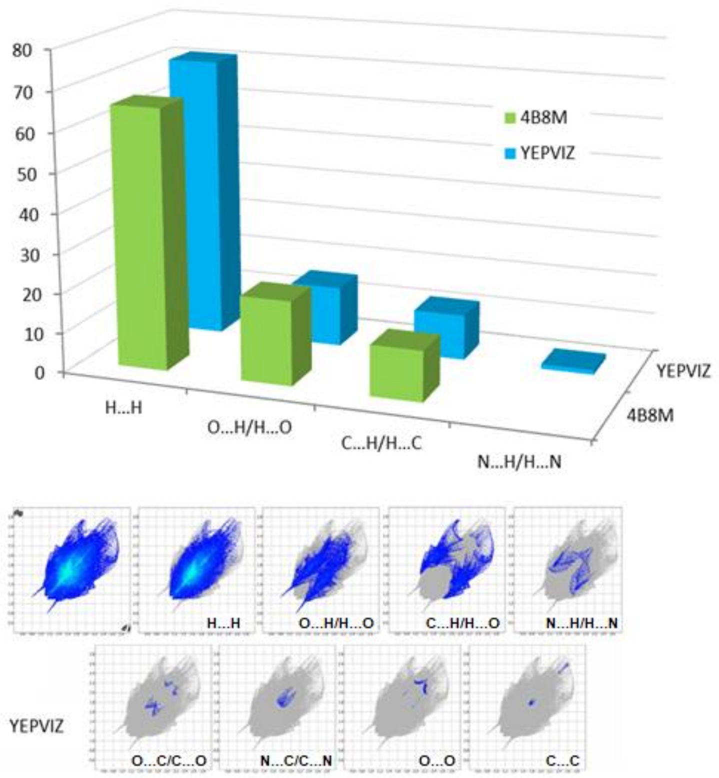

Figure S8. Comparison of inter-contacts contribution into both crystals (> 0.5 %) and fingerprint plots of YEPVIZ.

					E_ele	E_pol	E_dis	E_rep	E_tot
	0	x+1/2, -y+1/2, -z	11.56	B3LYP/ 6-31G(d,p)	-9.2	-2.9	-43.5	22.1	-36.0
	1	-x, -y, z	11.55	B3LYP/ 6-31G(d,p)	-2.7	-1.8	-14.9	7.1	-12.8
	1	-x, -y, z	9.77	B3LYP/ 6-31G(d,p)	10.2	-4.4	-67.4	29.8	-32.8
	2	x+1/2, -y+1/2, -z	11.49	B3LYP/ 6-31G(d,p)	-10.1	-1.9	-36.1	14.8	-34.4
	0	-x+1/2, y+1/2, -z	17.05	B3LYP/ 6-31G(d,p)	-9.7	-2.1	-16.8	0.0	-26.5
	2	-x+1/2, y+1/2, -z	17.00	B3LYP/ 6-31G(d,p)	-0.4	-0.7	-17.2	0.0	-16.0
	0	-x, -y, z	19.96	B3LYP/ 6-31G(d,p)	2.5	-0.2	-3.3	0.0	-0.4
	0	-x, -y, z	20.23	B3LYP/ 6-31G(d,p)	-0.4	-0.1	-2.1	0.0	-2.3

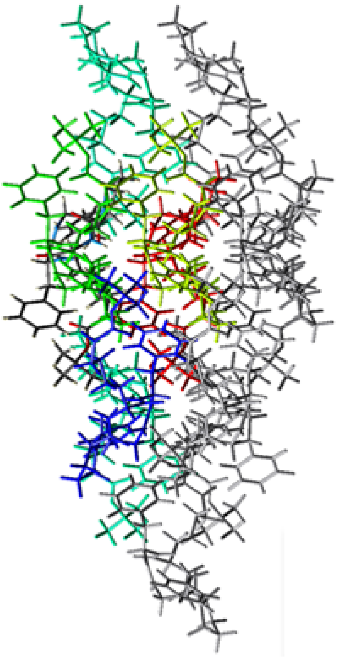

Scale factors for benchmarked energy models
See Mackenzie et al. IUCrJ (2017)

Energy Model	k_ele	k_pol	k_disp	k_rep
CE-HF ... HF/3-21G electron densities	1.019	0.651	0.901	0.811
CE-B3LYP ... B3LYP/6-31G(d,p) electron densities	1.057	0.740	0.871	0.618

Figure S9. Interaction energies of the molecular pairs in terms of the energy frameworks of 4B8M (scale factors for benchmarked energy models).

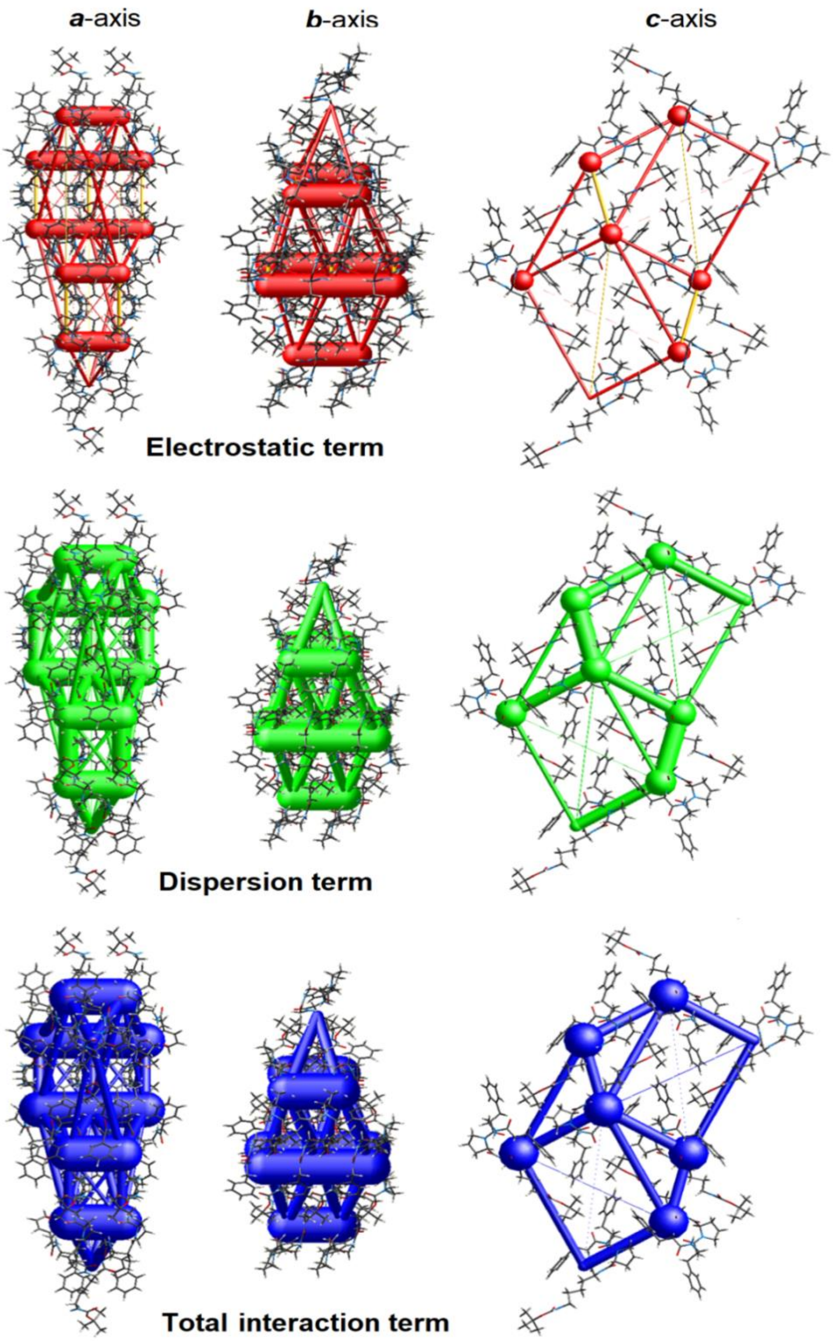

Figure S10. Energy frameworks of YEPVIZ corresponding to the electrostatic and dispersion energy components, and the total energy framework along *a*, *b* and *c*-axis. The cylinders thickness shows the relative strength of molecular packing.

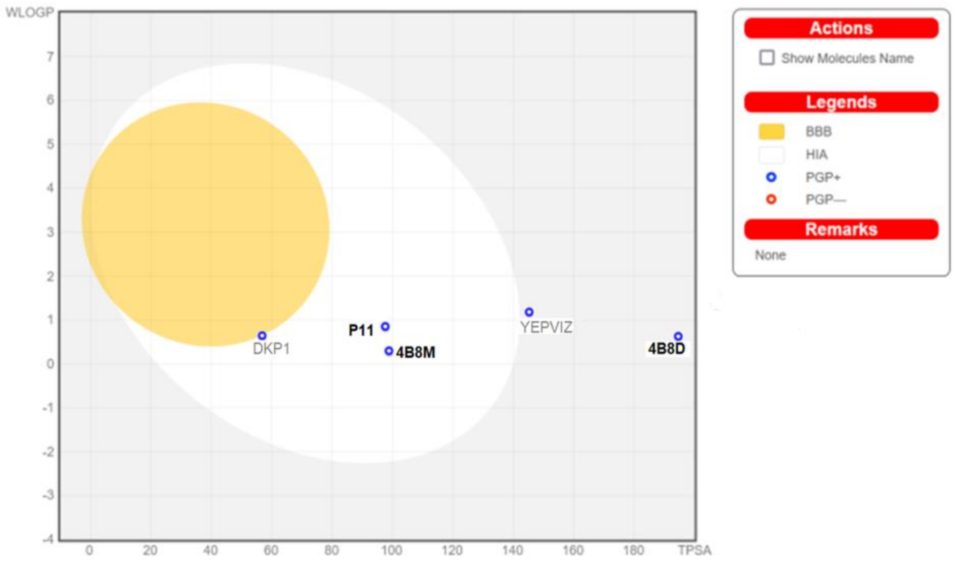

Figure S11. Predicted BOILED-Egg diagram of the studied peptides, from SwissADME web tool. *CLA – outside area of boiled egg.

Peptide	Prediction	Probability	Result	Structure
P11	Potential cardiotoxic (+)	50%	Yes (Value= 0.28 and limit = 0.26)	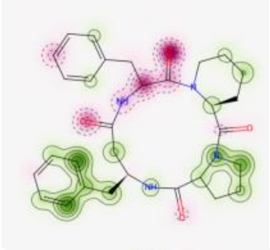
	Strong or Extreme	50%		
CLA	Potential cardiotoxic (+)	50%	Yes (Value= 0.27 and limit = 0.26)	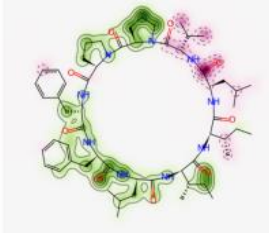
	Strong or Extreme	60%		
4B8D	Non-cardiotoxic (-)	50%	Yes (Value= 0.28 and limit = 0.26)	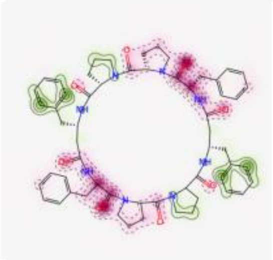
	Not applicable	Not applicable		
DKP1	Non-cardiotoxic (-)	50%	No (Value= 0.25 and limit = 0.26)	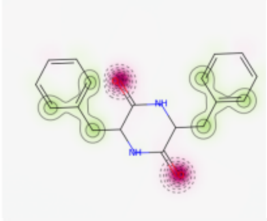
	Not applicable	Not applicable		
YEPVIZ	Potential cardiotoxic (+)	50%	Yes (Value= 0.28 and limit = 0.26)	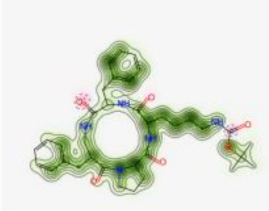
	Strong or Extreme	50%		

Figure S12. Maps of cardiac toxicity in selected compounds.

Table S1. Mutation status of key genes involved in cancer-related signaling pathways in DMBC29 cells, for which the PolyPhen-2-based functional effect of a mutation was predicted as possibly/probably damaging [73]. Prediction of functional effects of amino acid substitutions were assessed by using Polyphen-2 software and mutations were marked as homozygous (+/+) or heterozygous (+/-).

**DMBC29**	
**gene**	**RTK/RAS signaling pathway**
BRAF	V600E+/- probably damaging0.971
ERBB2	P1170A +/+ possibly damaging 0.953
FGFR3	P451S +/- possibly damaging 0.902
FGFR4	G388R +/- probably damaging 0.998
**gene**	**WNT/β-catenin pathway**
DKK2	R146+/- probably damaging 1.000
RNF43	L418M +/+ probably damaging 0.969
**gene**	**HIPPO pathway**
DCHS2	frameshift variant +/+ N1365X
WWC1	Inframe deletion +/- E865del
**gene**	**MYC pathway**
MGA	P1523A +/+ possibly damaging 0.657
**gene**	**NOTCH pathway**
NCOR2	Inframe insertion +/+1849-1850/SSG; Inframe insertion +/- P511QP

Table S2. Mutation status of key genes involved in cancer-related signaling pathways in DMBC28 cells, for which the PolyPhen-2-based functional effect of a mutation was predicted as possibly/probably damaging [82]. Prediction of functional effects of amino acid substitutions were assessed by using Polyphen-2 software and mutations were marked as homozygous (+/+) or heterozygous (+/-).

**DMBC28**	
**gene**	**RTK/RAS signaling pathway**
BRAF	V600E+/- probably damaging0.971
ERBB2	P1170A +/+ possibly damaging 0.953
FGFR3	P451S +/- possibly damaging 0.902
FGFR4	G388R +/- probably damaging 0.998
NF1	R135W +/- probably damaging 1.000
**gene**	**WNT/β-catenin pathway**
RNF43	L418M +/+ probably damaging 0.969
DKK2	R146+/- probably damaging 1.000
**gene**	**HIPPO pathway**
CRB2	G159A +/- possibly damaging 0.610
DCHS2	frameshift variant +/+ N1365X
WWC1	Inframe deletion +/- E865del
**gene**	**MYC pathway**
MGA	P1523A +/+ possibly damaging 0.657
**gene**	**NOTCH pathway**
NCOR2	Inframe insertion +/+1849-1850/SSG; Inframe insertion +/- P511QP

Table S3. Molecular weights of short peptides and DMSO volume necessary to prepare 20 mM stock solutions.

	Symbol	Sequence of amino acids	Molecular weight [g/mol]	Mass [mg]	DMSO [µl]
1	4B8D	cyclo(Pro-Pro-*β*³*homo*Phe-Phe-Pro-Pro-*β*³*homo*Phe-Phe-)	987.3	1.9	96.2
2	4B8M-12	cyclo(Pro-Pro-*β*³*homo*Phe-Phe-)	502.6	2.7	268.6
3	4B8M-14	cyclo(Pro-Pro-*β*³*homo*Phe-Phe-)	502.6	3.4	338.3
4	P0011-6	cyclo(Pro-Pro-*β*³*homo*Phe-Phe-)	502.6	7.3	726.2
5	P0010-3-3	cyclo(Pro-Pro-*β*³*homo*Phe-Phe-)	502.6	5.1	507.4
6	DKP1	cyclo(Phe-Phe-)	294.4	3.4	577.4
7	P11	cyclo(Pro-*homo*Pro-*β*³*homo*Phe-Phe-)	516.6	2.0	193.5
8	P11L	H-Pro-*homo*Pro-*β*³*homo*Phe-Phe-OH xTFA	648	2.8	216
9	P03L	H-*D*-Pro-Pro-*β*³*homo*Phe-Phe-OH x TFA	634.7	2.1	165.4
10	P04L	H-Pro-Pro-*D*-*β*³*homo*Phe-Phe-OH x TFA	634.7	4.3	339
11	CLA	cyclo(Leu-Ile-Ile-Leu-Val-Pro-Pro-Phe-Phe-)	1040.4	1	48

Table S4. Relevant structure data of the tetrapeptide ring in YEPVIZ (see [66] for atom notation).

Bond length [Å]	X-ray	B3LYP	
		vacuum	solution
N3-C8	1.436(15)	1.474	1.476
C8-C3	1.519(17)	1.542	1.537
C3-N2	1.335(14)	1.355	1.349
N2-C1	1.448(14)	1.465	1.461
C1-C4	1.530(16)	1.548	1.546
C4-N1	1.340(15)	1.360	1.353
N1-C2	1.460(14)	1.458	1.459
C2-C12	1.495(19)	1.551	1.553
C12-N4	1.361(19)	1.360	1.352
N4-C10	1.445(16)	1.456	1.460
C10-C5	1.593(16)	1.572	1.569
C5-C9	1.533(17)	1.524	1.526
C9-N3	1.324(15)	1.361	1.353
N3-C17	1.466(15)	1.471	1.476
C17-C18	1.548(2)	1.531	1.531
C18-C7	1.508(2)	1.538	1.537
C7-C8	1.544(2)	1.547	1.545
C3-O2	1.230(15)	1.226	1.233
C4-O3	1.214(15)	1.220	1.229
C12-O4	1.235(17)	1.223	1.229
C9-O1	1.240(16)	1.235	1.240

Table S4. Cont.

Bond angle [°]	X-ray	B3LYP	
		vacuum	solution
C9-N3-C8	118.2(9)	118.8	119.2
N3-C8-C3	108.5(9)	108.4	108.4
C8-C3-N2	117.0(10)	117.4	117.3
C3-N2-C1	119.6(9)	121.4	121.6
N2-C1-C4	115.6(9)	114.5	113.7
C1-C4-N1	115.4(10)	116.9	117.1
C4-N1-C2	122.0(9)	123.0	123.8
N1-C2-C12	111.0(10)	111.1	111.4
C2-C12-N4	118.2(12)	115.6	115.7
C12-N4-C10	123.5(11)	123.4	124.2
N4-C10-C5	112.3(9)	114.2	114.0
C10-C5-C9	106.2(9)	107.7	107.5
C5-C9-N3	119.4(10)	118.9	118.7
C9-N3-C17	126.5(10)	128.9	128.7

C8-N3-C17	115.1(9)	112.0	112.1
N3-C17-C18	101.3(10)	102.4	102.4
C17-C18-C7	102.6(11)	103.2	103.4
C18-C7-C8	106.3(10)	104.5	104.2
C7-C8-N3	101.9(9)	103.9	104.0
C7-C8-C3	113.7(10)	111.4	112.0
C8-C3-O2	119.9(11)	120.1	120.7
N2-C3-O2	123.1(11)	122.6	122.0
C1-C4-O3	119.4(11)	118.9	119.0
N1-C4-O3	125.2(11)	124.2	123.9
C2-C12-O4	120.4(12)	121.1	120.8
N4-C12-O4	121.4(13)	123.3	123.5
C5-C9-O1	118.1(11)	121.0	120.5
N3-C9-O1	122.1(12)	119.8	120.4

Table S4. Cont.

Torsion angle [°]	X-ray		B3LYP
		vacuum	solution
N3-C8-C3-N2	-131.4(10)	-127.5	-127.8
C8-C3-N2-C1	175.1(10)	177.9	178.9
C3-N2-C1-C4	-54.6(13)	-64.7	-61.1
N2-C1-C4-N1	-29.0(14)	-22.8	-27.1
C1-C4-N1-C2	178.7(9)	176.2	178.0
C4-N1-C2-C12	-127.2(11)	-125.1	-125.6
N1-C2-C12-N4	33.6(15)	29.5	29.4
C2-C12-N4-C10	-164.8(11)	-161.5	-163.6
C12-N4-C10-C5	84.0(14)	82.7	86.1
N4-C10-C5-C9	36.4(12)	38.4	37.2
C10-C5-C9-N3	84.3(12)	86.6	85.8
C5-C9-N3-C8	-168.8(10)	-162.6	-163.2
C9-N3-C8-C3	62.7(13)	60.0	60.7
C5-C9-N3-C17	15.2(17)	11.0	15.7
C9-N3-C17-C18	155.4(11)	160.6	157.9
N3-C17-C18-C7	33.0(13)	36.2	35.6
C17-C18-C7-C8	-35.1(13)	-34.5	-35.8
C18-C7-C8-N3	22.7(12)	19.2	21.7
C18-C7-C8-C3	139.2(11)	135.7	138.6
C7-C8-C3-N2	116.0(11)	118.7	118.0
N3-C8-C3-O2	47.5(14)	52.2	50.9
C1-N2-C3-O2	-3.7(17)	-1.8	0.3
N2-C1-C4-O3	151.9(10)	158.0	153.9
C2-N1-C4-O3	-2.3(17)	-4.6	-3.1
N1-C2-C12-O4	-146.5(12)	-149.9	-151.3
C10-N4-C12-O4	15.3(19)	17.9	17.1
C10-C5-C9-O1	-88.4(12)	-87.5	-87.5
C8-N3-C9-O1	3.7(16)	11.6	10.0

Table S5. NBO atomic charges of YEPVIZ (see [66] for atom notation).

Atom	vacuum	solvent
O1	-0.685	-0.712
N1	-0.672	-0.659
O2	-0.658	-0.698
N2	-0.629	-0.620
N3	-0.505	-0.493
O3	-0.632	-0.691
O4	-0.652	-0.686
N4	-0.647	-0.639
C1	-0.092	-0.088
C2	-0.094	-0.091
C3	0.685	0.697

C4	0.691	0.704
C5	-0.287	-0.287
C6	-0.396	-0.398
C7	-0.383	-0.380
C8	-0.072	-0.073
C9	0.719	0.719
C10	-0.172	-0.174
C11	-0.033	-0.032
C12	0.694	0.697
C13	-0.207	-0.216
C14	-0.028	-0.033
O5	-0.612	-0.613
C15	-0.194	-0.206
C16	-0.209	-0.209
C17	-0.164	-0.165
C18	-0.384	-0.385
C19	-0.398	-0.401
C20	0.277	0.279
C21	-0.203	-0.206
C22	-0.379	-0.378
C23	-0.210	-0.218
C24	-0.203	-0.206
C25	-0.187	-0.206
C26	-0.201	-0.206
N5	-0.650	-0.641
C27	-0.209	-0.219
C28	-0.192	-0.206
C29	-0.379	-0.381
O6	-0.667	-0.712
C30	-0.579	-0.584
C31	-0.605	-0.606
C32	-0.604	-0.606
C33	-0.174	-0.174
C34	-0.385	-0.384
C35	0.924	0.929

Table S6. Relevant MO energies (in eV) of the systems under study.

	4B8M		YEPVIZ	
	vacuum	solution	vacuum	solution
LUMO	-0.721	-0.693	-0.843	-0.632
HOMO	-6.648	-6.808	-6.479	-6.794
HOMO-1	-6.803	-6.931	-6.708	-6.854
HOMO-2	-6.879	-7.018	-6.801	-7.082
HOMO-3	-6.915	-7.031	-6.914	-7.112
HOMO-LUMO energy gap	5.926	6.115	5.636	6.163

Table S7. Selected bond and angles in crystal structure 4B8M (see Fig. 9 for atom notation).

O4-C30	1.231(2)	C3-C4	1.534(3)	C30-N1-C4	130.16(16)	C9-N2-C5-O1	-176.2(2)
O1-C5	1.221(2)	C24-C25	1.386(3)	C4-N1-C1	109.82(16)	C6-N2-C5-O1	-15.0(3)
N1-C30	1.349(3)	C24-C23	1.392(3)	C5-N2-C9	126.99(17)	C9-N2-C5-C4	-1.9(3)
N1-C4	1.471(2)	C23-C22	1.511(3)	C20-N4-C29	119.84(18)	C6-N2-C5-C4	159.31(19)
N1-C1	1.476(3)	C22-C29	1.544(3)	C10-N3-C11	122.54(18)	C30-N1-C1-C2	-155.90(18)
N2-C5	1.358(3)	C27-C26	1.386(3)	O1-C5-N2	121.47(18)	C4-N1-C2-C1	11.8(2)
N2-C9	1.472(3)	C29-C30	1.533(3)	N2-C5-C4	117.78(17)	C30-N1-C4-C3	132.7(2)
N2-C6	1.474(3)	C14-C15	1.385(4)	C27-C28-C23	120.75(19)	C1-N1-C4-C3	-33.06(18)
O3-C20	1.227(3)	C14-C13	1.390(3)	C2-C3-C4	102.59(16)	C30-N1-C4-C5	-113.6(2)
N4-C20	1.361(3)	C20-C19	1.515(3)	N1-C4-C5	108.82(15)	C1-N1-C4-C5	80.6(2)
N4-C29	1.455(3)	C9-C10	1.512(4)	C28-C23-C24	118.2(2)	C2-C3-C4-N1	40.80(18)
O2-C10	1.231(3)	C9-C8	1.546(3)	C24-C23-C22	121.2(2)	C2-C3-C4-C5	-73.46(18)
N3-C10	1.335(3)	C12-C13	1.509(3)	N4-C29-C30	114.97(15)	O1-C5-C4-N1	-16.2(3)
N3-C11	1.449(3)	C12-C11	1.540(3)	C30-C29-C22	109.71(16)	N2-C5-C4-N1	169.44(18)

C5-C4	1.548(3)	C26-C25	1.377(4)	O4-C30-N1	119.82(18)	O1-C5-C4-C3	93.0(2)
C1-C2	1.532(3)	C6-C7	1.511(4)	N1-C30-C29	120.09(16)	N2-C5-C4-C3	-81.4(2)
C28-C27	1.386(3)	C13-C18	1.392(3)	O3-C20-N4	122.8(2)	C27-C28-C23-C22	-179.45(18)
C28-C23	1.389(3)	C19-C11	1.539(4)	O2-C10-N3	124.9(3)	C25-C24-C23-C22	178.22(18)
C3-C2	1.532(3)	C18-C17	1.384(4)	N2-C6-C7	102.7(2)	C6-N2-C9-C10	114.6(2)

Table S8. X-H/O···π interactions in 4B8M.

	H/O···π	X···π	X –H/O···π
C7-H7A···π ^{i}	2.88	3.714(3)	145
C20-O3···π ^{ii}	3.688(2)	3.856(2)	72.97(13)

(i) -1+x, 1+y, -1+z; (ii) x, y, z

Table S9. Supramolecular synthon patterns (< 20-membered motifs) in 4B8M and YEPVIZ.

**4B8M**	
C(4)	$_{(NH)}$N3-H3N···O2$_{(C=O)}$
C(5)	$_{\textbf{(CH-Pro)}}$**C3-H3B**···O1$_{(C=O)}$<br>$_{\textbf{(CH-Pro)}}$**C9-H9**···O1$_{(C=O)}$
C(6)	$_{\textbf{(CH-Pro)}}$**C3-H3A**···O4$_{(C=O)}$<br>$_{(CH)}$C19-H19B···O2$_{(C=O)}$
C(8)	$_{(NH)}$N4-H4N···O2$_{(C=O)}$<br>$_{(CH)}$C28-H28···O3$_{(C=O)}$
C(9)	$_{(CH)}$C26-H26···O4$_{(C=O)}$
C(13)	$_{(CH)}$C16-H16···O4$_{(C=O)}$
$R^1_2(8)$	$_{(NH)}$N3-H3N···O2$_{(C=O)}$ & $_{(NH)}$N4-H4N···O2$_{(C=O)}$
Level 2	
$C^1_2(8)$	$_{(CH)}$C16-H16···O4$_{(C=O)}$ & $_{(CH)}$C26-H26···O4$_{(C=O)}$
$C^1_2(13)$	$_{\textbf{(CH-Pro)}}$**C3-H3A**···O4$_{(C=O)}$ & $_{(CH)}$C26-H26···O4$_{(C=O)}$
$C^2_2(10)$	$_{(NH)}$N3-H3N···O2$_{(C=O)}$ & $_{(CH)}$C19-H19B···O2$_{(C=O)}$<br>$_{\textbf{(CH-Pro)}}$**C3-H3B**···O1$_{(C=O)}$ & $_{\textbf{(CH-Pro)}}$**C3-H3A**···O4$_{(C=O)}$<br>$_{\textbf{(CH-Pro)}}$**C3-H3B**···O1$_{(C=O)}$ & $_{\textbf{(CH-Pro)}}$**C9-H9**···O1$_{(C=O)}$
$C^2_2(11)$	$_{(NH)}$N3-H3N···O2$_{(C=O)}$ & $_{\textbf{(CH-Pro)}}$**C9-H9**···O1$_{(C=O)}$<br>$_{(CH)}$C26-H26···O4$_{(C=O)}$ & $_{(CH)}$C28-H28···O3$_{(C=O)}$<br>$_{\textbf{(CH-Pro)}}$**C3-H3B**···O1$_{(C=O)}$ & $_{\textbf{(CH-Pro)}}$**C3-H3A**···O4$_{(C=O)}$
$C^2_2(13)$	$_{\textbf{(CH-Pro)}}$**C3-H3A**···O4$_{(C=O)}$ & $_{\textbf{(CH-Pro)}}$**C9-H9**···O1$_{(C=O)}$<br>$_{\textbf{(CH-Pro)}}$**C9-H9**···O1$_{(C=O)}$ & $_{(CH)}$C19-H19B···O2$_{(C=O)}$
$C^2_2(14)$	$_{(NH)}$N4-H4N···O2$_{(C=O)}$ & $_{\textbf{(CH-Pro)}}$**C3-H3B**···O1$_{(C=O)}$<br>$_{(NH)}$N4-H4N···O2$_{(C=O)}$ & $_{(CH)}$C19-H19B···O2$_{(C=O)}$
$C^2_2(15)$	$_{(NH)}$N4-H4N···O2$_{(C=O)}$ & $_{\textbf{(CH-Pro)}}$**C9-H9**···O1$_{(C=O)}$<br>$_{(NH)}$N4-H4N···O2$_{(C=O)}$ & $_{(CH)}$C16-H16···O4$_{(C=O)}$<br>$_{(NH)}$N3-H3N···O2$_{(C=O)}$ & $_{\textbf{(CH-Pro)}}$**C3-H3B**···O1$_{(C=O)}$<br>$_{\textbf{(CH-Pro)}}$**C3-H3A**···O4$_{(C=O)}$ & $_{(CH)}$C26-H26···O4$_{(C=O)}$
$C^2_2(16)$	$_{(NH)}$N4-H4N···O2$_{(C=O)}$ & $_{(CH)}$C28-H28···O3$_{(C=O)}$<br>$_{\textbf{(CH-Pro)}}$**C3-H3A**···O4$_{(C=O)}$ & $_{(CH)}$C28-H28···O3$_{(C=O)}$<br>$_{(CH)}$C19-H19B···O2$_{(C=O)}$ & $_{(CH)}$C28-H28···O3$_{(C=O)}$<br>$_{\textbf{(CH-Pro)}}$**C3-H3B**···O1$_{(C=O)}$ & $_{(CH)}$C19-H19B···O2$_{(C=O)}$<br>$_{\textbf{(CH-Pro)}}$**C3-H3A**···O4$_{(C=O)}$ & $_{(CH)}$C16-H16···O4$_{(C=O)}$
$C^2_2(17)$	$_{(NH)}$N4-H4N···O2$_{(C=O)}$ & $_{\textbf{(CH-Pro)}}$**C3-H3A**···O4$_{(C=O)}$<br>$_{(CH)}$C26-H26···O4$_{(C=O)}$ & $_{(CH)}$C28-H28···O3$_{(C=O)}$<br>$_{(CH)}$C16-H16···O4$_{(C=O)}$ & $_{(CH)}$C28-H28···O3$_{(C=O)}$<br>$_{\textbf{(CH-Pro)}}$**C9-H9**···O1$_{(C=O)}$ & $_{(CH)}$C16-H16···O4$_{(C=O)}$<br>$_{(CH)}$C16-H16···O4$_{(C=O)}$ & $_{(CH)}$C19-H19B···O2$_{(C=O)}$
$C^2_2(18)$	$_{(NH)}$N4-H4N···O2$_{(C=O)}$ & $_{(CH)}$C26-H26···O4$_{(C=O)}$<br>$_{(NH)}$N3-H3N···O2$_{(C=O)}$ & $_{(CH)}$C28-H28···O3$_{(C=O)}$<br>$_{(NH)}$N3-H3N···O2$_{(C=O)}$ & $_{\textbf{(CH-Pro)}}$**C3-H3A**···O4$_{(C=O)}$<br>$_{(NH)}$N3-H3N···O2$_{(C=O)}$ & $_{(CH)}$C16-H16···O4$_{(C=O)}$<br>$_{\textbf{(CH-Pro)}}$**C3-H3B**···O1$_{(C=O)}$ & $_{(CH)}$C26-H26···O4$_{(C=O)}$<br>$_{(CH)}$C16-H16···O4$_{(C=O)}$ & $_{(CH)}$C19-H19B···O2$_{(C=O)}$
$C^2_2(19)$	$_{(NH)}$N4-H4N···O2$_{(C=O)}$ & $_{(CH)}$C26-H26···O4$_{(C=O)}$<br>$_{(NH)}$N3-H3N···O2$_{(C=O)}$ & $_{(CH)}$C16-H16···O4$_{(C=O)}$<br>$_{\textbf{(CH-Pro)}}$**C3-H3B**···O1$_{(C=O)}$ & $_{(CH)}$C28-H28···O3$_{(C=O)}$<br>$_{\textbf{(CH-Pro)}}$**C3-H3B**···O1$_{(C=O)}$ & $_{(CH)}$C16-H16···O4$_{(C=O)}$

	(CH-Pro)**C3-H3A**···O4(C=O) & (CH)C19-H19B···O2(C=O)<br>(CH-Pro)**C3-H3A**···O4(C=O) & (CH)C16-H16···O4(C=O)
$C^2_2(20)$	(NH)N4-H4N···O2(C=O) & (CH)C16-H16···O4(C=O)<br>(CH-Pro)**C9-H9**···O1(C=O) & (CH)C26-H26···O4(C=O)<br>(CH)C19-H19B···O2(C=O) & (CH)C26-H26···O4(C=O)
$R^1_2(6)$	(NH)N4-H4N···O2(C=O) & (CH)C19-H19B···O2(C=O)<br>(NH)N3-H3N···O2(C=O) & (CH)C19-H19B···O2(C=O)
$R^1_2(8)$	(CH-Pro)**C3-H3B**···O1(C=O) & (CH-Pro)**C9-H9**···O1(C=O)
$R^2_2(9)$	(CH-Pro)**C3-H3B**···O1(C=O) & (CH-Pro)**C3-H3A**···O4(C=O)
$R^2_2(11)$	(NH)N3-H3N···O2(C=O) & (CH-Pro)**C9-H9**···O1(C=O)
$R^2_2(12)$	(NH)N4-H4N···O2(C=O) & (CH-Pro)**C9-H9**···O1(C=O)
$R^2_2(13)$	(NH)N4-H4N···O2(C=O) & (CH-Pro)**C3-H3A**···O4(C=O)<br>(CH-pro)**C3-H3A**···O4(C=O) & (CH-Pro)**C9-H9**···O1(C=O)
$R^2_2(14)$	(NH)N4-H4N···O2(C=O) & (CH)C28-H28···O3(C=O)
$R^2_2(15)$	(NH)N3-H3N···O2(C=O) & (CH-Pro)**C3-H3B**···O1(C=O)<br>(CH-Pro)**C3-H3A**···O4(C=O) & (CH)C19-H19B···O2(C=O)
$R^2_2(16)$	(NH)N4-H4N···O2(C=O) & (CH-Pro)**C3-H3B**···O1(C=O)<br>(CH-Pro)**C3-H3A**···O4(C=O) & (CH)C28-H28···O3(C=O)<br>(CH)C19-H19B···O2(C=O) & (CH)C28-H28···O3(C=O)
$R^2_2(17)$	(NH)N3-H3N···O2(C=O) & (CH-Pro)**C3-H3A**···O4(C=O)<br>(CH-Pro)**C3-H3B**···O1(C=O) & (CH)C19-H19B···O2(C=O)
$R^2_2(18)$	(NH)N3-H3N···O2(C=O) & (CH)C28-H28···O3(C=O)<br>(CH-Pro)**C9-H9**···O1(C=O) & (CH)C28-H28···O3(C=O)
$R^2_2(19)$	(CH)C3-H3B···O1(C=O) & (CH)C28-H28···O3(C=O)<br>(CH-Pro)**C9-H9**···O1(C=O) & (CH)C19-H19B···O2(C=O)
**YEPVIZ**	
S(6)	(CH)C34-H37···O6(C=O)
S(7)	(CH)C6-H7···O4(C=O)
S(9)	(NH)N1-H1···O1(C=O)<br>(NH)N4-H3···O2(C=O)
C(5)	(NH)N2-H2···O3(C=O)
C(7)	(CH-Pro)**C8-H8**···O4(C=O)<br>(CH-Pro)**C8-H11**···O3(C=O)
C(8)	(CH)C2-H5···O1(C=O)
Level 2	
$C^2_2(12)$	(NH)N2-H2···O3(C=O) & (CH-Pro)**C8-H11**···O3(C=O)
$C^2_2(15)$	(NH)C2-H5···O1(C=O) & (CH-Pro)**C8-H8**···O4(C=O)
$C^2_2(16)$	(NH)N2-H2···O3(C=O) & (CH)C2-H5···O1(C=O)
$C^2_2(18)$	(NH)N2-H2···O3(C=O) & (CH)C6-H8···O4(C=O)<br>(NH-Pro)**C8-H11**···O3(C=O) & (CH)C2-H5···O1(C=O)
$C^2_2(19)$	(CH-Pro)**C8-H8**···O4(C=O) & (CH-Pro)**C8-H11**···O3(C=O)
$R^1_2(6)$	(NH)N2-H2···O3(C=O) & (CH-Pro)**C8-H11**···O3(C=O)
$R^2_2(9)$	(NH)C2-H5···O1(C=O) & (CH-Pro)**C8-H8**···O4(C=O)
$R^2_2(10)$	(CH-Pro)**C8-H11**···O3(C=O) & (CH)C2-H5···O1(C=O)
$R^2_2(12)$	(NH)N2-H2···O3(C=O) & (CH)C2-H5···O1(C=O)<br>(NH)N2-H2···O3(C=O) & (CH)C6-H8···O4(C=O)
$R^2_2(13)$	(CH-Pro)**C8-H8**···O4(C=O) & (CH-Pro)**C8-H11**···O3(C=O)

Table S10. Major interactions, Hirshfeld contact surfaces and enrichment ratio (ER) for 4B8M and YEPVIZ (for > 0.5).

Interactions	4B8M	YEPVIZ						
Surface (%)	H			O		C		N
	82.75	85.05	10.65	7.6	6.4	5.7	-	0.6
Major contacts	H···H			O···H		C···H		N···H
Proportion (%)	65.7	71.1	65.7	15.2	12.8	11.5	-	1.2
ER	0.96	1.17	1.21	1.17	1.21	1.17	-	1.17

Table S11. ADMET parameters in synthesized compounds.

**Physicochemical properties**

Peptide ranker score	formula	Mol. weight [g/mol]	Num. Heavy atoms	Num. Arom. Heavy atoms	Fraction Csp³	Num. Rotatable bonds	Num. H-bond acceptors	Num. H-bonds donors	Molar refractivity	TPSA* [Å²]
4B8M *0.98292*	$C_{29}H_{34}N_4O_4$	502.60	37	12	0.45	4	4	2	154.32	98.82
P11 *0.9469*	$C_{30}H_{36}N_4O_4$	516.63	38	12	0.47	4	4	2	159.13	98.82
CLA *0.91503*	$C_{57}H_{85}N_9O_9$	1040.34	75	12	0.63	13	9	7	323.50	244.32
4B8D *0.01823*	$C_{58}H_{68}N_8O_8$	1005.21	74	24	0.45	8	8	4	308.64	197.64
DKP1 *0.98537*	$C_{18}H_{18}N_2O_2$	294.35	22	12	0.22	4	2	2	91.65	58.20
YEPVIZ	$C_{35}H_{47}N_5O_6$	633.78	46	12	0.51	12	6	4	189.70	145.94

*TPSA - topological polar surface area

**Lipophilicity**						
	Log $P_{o/w}$ (iLOGP)	Log $P_{o/w}$ (XLOGP3)	Log $P_{o/w}$ (WLOGP)	Log $P_{o/w}$ (MLOGP)	Log $P_{o/w}$ (SILICOS-IT)	Consensus log $P_{o/w}$
4B8M	3.88	2.71	0.30	1.70	2.32	2.18
P11	3.27	3.07	0.69	1.89	2.56	2.30
CLA	6.15	7.76	0.27	0.59	4.88	3.93
4B8D	5.24	5.42	0.61	1.45	3.51	3.25
DKP1	2.23	1.59	0.69	1.93	2.98	1.89
YEPVIZ	3.61	3.78	1.35	1.65	3.56	2.79

LogP represents the n-octanol/water *partition* coefficient
MLogP - Moriguchi model of octanol-water partition coefficient

**Water solubility**									
	Log S (ESOL)	Solubility [mg/ml; mol/l]	class	Log S (Ali)	Solubility [mg/ml; mol/l]	class	Log S (SILICOS-IT)	Solubility [mg/ml; mol/l]	class
4B8M	-4.64	1.15e-02; 2.29e-05	moderately soluble	-4.44	1.83e-0.2; 3.64e-05	Moderately soluble	-7.12	3.85e-0.5; 7.66e-0.8	poorly soluble
P11	-4.95	5.84e-03; 1.13e-05	moderately soluble	-4.81	7.96e-03; 1.54e-05	Moderately soluble	-7.38	2.16e-05; 4.18e-08	poorly soluble
CLA	-10.44	3.78e-08; 3.64e-11	insoluble	-12.73	1.92e-10; 1.84e-13	insoluble	-13.07	8.75e-11; 8.41e-14	insoluble
4B8D	-9.20	6.36e-07; 6.33e-10	poorly soluble	-9.33	4.75e-07; 4.72e-10	poorly soluble	-13.77	1.71e-11; 1.71e-14	insoluble
DKP1	-2.81	4.60e-01; 1.56e-03	soluble	-2.42	1.11e+00; 3.77e-03	soluble	-6.24	1.68e-04; 5.70e-07	poorly soluble
YEPVIZ	-5.55	1.78e-0.4; 2.89e-0.7	moderately soluble	-6.54	1.83e-0.4; 2.89e-0.7	poorly soluble	-9.40	2.49e-0.7; 3.94e-10	poorly soluble

**Pharmacokinetics**									
	GI absorption	BBB permeant	P-gp substrate	CYP1A2 inhibitor	CYP2C19 inhibitor	CYP2C9 inhibitor	CYP2D6 inhibitor	CYP3A4 inhibitor	Log *Kp* (skin permeation) [cm/s]
4B8M	high	no	yes	no	no	no	yes	yes	-7.44
P11	high	no	yes	no	no	no	no	yes	-7.27
CLA	low	no	yes	no	no	no	no	no	-7.14
4B8D	low	no	yes	no	no	no	no	yes	-8.58
DKP1	high	no	yes	no	no	no	yes	no	-6.97
YEPVIZ	low	no	yes	no	no	no	no	yes	-7.48

GI - gastrointestinal absorption, *BBB* - blood–brain barrier penetration, CYP - cytochrome P450

**Druglikeness**						
	Lipinski	Ghose	Veber	Egan	Muegge	Bioavailability score
4B8M	yes, 1 violation	no, 3 violations	yes	yes	yes	0.55
P11	yes, 1 violation	no, 3 violations	yes	yes	yes	0.55
CLA	no, 3 violations	no, 3 violations	no, 2 violations	no, 1 violation	no, 4 violations	0.17
4B8D	no, 2 violations	no, 3 violations	no, 1 violation	no, 1 violation	no, 4 violations	0.17
DKP1	no, 0 violation	yes	yes	yes	yes	0.55

YEPVIZ	no, 2 violations	no, 3 violations	no, 2 violations	no, 1 violation	no, 1 violation	0.17

Medicinal chemistry				
	PAINS	Brenk	Leadlikeness	Synthetic accessibility
4B8M	0 alert	0 alert	no, 1violation	4.85
P11	0 alert	0 alert	no, 1violation	5.00
CLA	0 alert	0 alert	no, 3 violations	9.08
4B8D	0 alert	0 alert	no, 3 violations	8.28
DKP1	0 alert	0 alert	yes	2.63
YEPVIZ	0 alert	0 alert	no, 3violation	5.96

PAINS pan-assay interference substructures

Table S12. ADMET parameters for studied peptides (from pkcsm web-tool).

Model name	4B8M	P11	CLA	4B8D	DKP1	YEPVIZ	unit
**ABSORPTION**							
Water solubility	-3.373	-3.438	-2.935	-2.905	-3.398	-4.176	log mol/L
Caco2 permeability	0.875	0.936	0.595	0.659	1.239	0.551	log Papp in 10^{-6} cm/s
Intestinal absorption (human)	74.328	75.16	39.327	53.449	96.366	59.305	% absorbed
Skin permeability	-2.805	-2.801	-2.735	-2.735	-2.887	-2.736	log Kp
P-glycoprotein substrate	yes	yes	yes	yes	yes	yes	yes/no
P-glycoprotein I inhibitor	yes	yes	yes	yes	no	yes	yes/no
P-glycoprotein II inhibitor	yes	yes	yes	yes	no	yes	yes/no
**DISTRIBUTION**							
VDss (human)	0.729	0.757	0.633	0.298	0.014	-0.076	log L/kg
Fraction unbound (human)	0.142	0.128	0.147	0.167	0.108	0	(Fu)
BBB permeability	-0.292	-0.315	-1.163	-0.501	0.08	-0.676	log BB
CNS permeability	-2.58	-2.489	-2.869	-3.246	-2.364	-3.083	log PS
**METABOLISM**							
CYP2D6 substrate	no	no	no	no	no	no	yes/no
CYP3A4 substrate	yes	yes	no	yes	yes	yes	yes/no
CYP1A2 inhibitor	no	no	no	no	yes	no	yes/no
CYP2C19 inhibitor	no	no	no	no	yes	no	yes/no
CYP2C9 inhibitor	no	no	no	no	no	no	yes/no
CYP2D6 inhibitor	no	no	no	no	no	no	yes/no
CYP3A4 inhibitor	yes	yes	no	yes	no	yes	yes/no
**EXCRETION**							
Total clearance	0.695	0.692	-0.017	-0.243	0.32	0.472	log ml/min/kg
Renal OCT2 substrate	yes	yes	no	no	no	no	yes/no
**TOXICITY**							
AMES toxicity	no	no	no	no	no	no	yes/no
max. tolerated dose (human)	-1.374	-1.379	0.466	0.418	-0.304	-0.719	log mg/kg/day
hERG I inhibitor	no	no	no	no	no	no	yes/no
hERG II inhibitor	yes	yes	yes	yes	yes	yes	yes/no
Oral rat acute toxicity (LD50)	2.504	2.532	2.628	2.547	2.127	3.653	mol/kg
Oral rat chronic toxicity (LOAEL)	1.896	1.765	5.027	3.4	1.374	1.694	log mg/kg_bw/day
hepatoxicity	yes	yes	yes	yes	no	yes	yes/no
Skin sensitisation	no	no	no	no	no	no	yes/no
T. Pyriformis toxicity	0.34	0.335	0.285	0.285	1.004	0.286	log ug/L
Minnow toxicity	3.312	3.195	10.603	5.38	0.625	3.788	log mM

Table S13. The probability values related to the confidence level of mutagenicity, carcinogenicity, hepatotoxicity and estrogenicity in relation to synthesized peptides.

	mutagenicity	carcinogenicity	hepatotoxicity	estrogenicity
4B8M	17	40	50	7
P11	25	36	51	7
CLA	14	40	75	8

4B8D	20	35	50	13
DKP1	21	33	60	5
YEPVIZ	26	38	65	6

Table S14. Cytotoxicity for tumor human cell lines in terms of analysed peptides.

	Pa	Pi	Cell-line	Cell-line full name	tissue	Tumor type
4B8M	0.546	0.016	A498	renal carcinoma	kidney	carcinoma
	0.526	0.013	BT-549	breast ductal carcinoma	breast	carcinoma
P11	0.526	0.018	A498	renal carcinoma	kidney	carcinoma
	0.514	0.014	BT-549	breast ductal carcinoma	breast	carcinoma
CLA	0.635	0.010	MDA-MB-231	breast adenocarcinoma	breast	adenocarcinoma
	0.581	0.005	NCI-H187	small cell lung carcinoma	lung	carcinoma
	0.557	0.014	SF-268	glioblastoma	brain	glioblastoma
	0.523	0.030	DMS-114	lung carcinoma	lung	carcinoma
4B8D	0.546	0.016	A498	renal carcinoma	kidney	carcinoma
	0.526	0.013	BT-549	breast ductal carcinoma	breast	carcinoma
DKP1	0.621	0.21	Hs683	oligodendroglioma	brain	glioma
	0.516	0.011	NCI-H187	small cel lung carcinoma	lung	carcinoma
YEPVIZ	0.560	0.019	MDA-MB-231	breast adenocarcinoma	breast	adenocarcinoma

Table S15. Bioactivity scores of the studied peptide molecules calculated on the basis of GPCR ligand, ion channel modulator, kinase inhibitor, nuclear receptor ligand, protease inhibitor, enzyme inhibitor interactions.

	GPCR ligand	Ion channel modulator	Kinase inhibitor	Nuclear receptor ligand	Protease inhibitor	Enzyme inhibitor
4B8M	0.33	0.02	-0.02	-0.00	0.52	0.12
P11	0.39	-0.01	-0.06	-0.16	0.58	0.14
CLA	-3.69	-3.80	-3.80	-3.81	-3.54	-3.74
4B8D	-3.67	-3.79	-3.79	-3.80	-3.54	-3.73
DKP1	0.21	0.09	-0.02	-0.05	0.33	0.09
YEPVIZ	0.20	-0.56	-0.48	-0.40	0.65	-0.21
